# DTX3L and ARTD9 inhibit IRF1 expression and mediate in cooperation with ARTD8 survival and proliferation of metastatic prostate cancer cells

**DOI:** 10.1186/1476-4598-13-125

**Published:** 2014-05-27

**Authors:** Samia B Bachmann, Sandra C Frommel, Rosalba Camicia, Hans C Winkler, Raffaella Santoro, Paul O Hassa

**Affiliations:** 1 Institute of Veterinary Biochemistry and Molecular Biology, University of Zurich, Winterthurerstrasse 190, 8057 Zurich, Switzerland; 2 Molecular Life Science Program, Life Science Zurich Graduate School, University of Zurich, Winterthurerstrasse 190, 8057 Zurich, Switzerland; 3 Stem Cell Research Laboratory, NHS Blood and Transplant, Nuffield Division of Clinical Laboratory Sciences, Radcliffe Department of Medicine, University of Oxford, Oxford OX3 9DU, UK; 4 Institute of Pharmacology and Toxicology, Vetsuisse Faculty, University of Zurich, Winterthurerstrasse 260, 8057 Zurich, Switzerland

**Keywords:** Metastatic prostate cancer, Mono-ADP-ribosyltransferase, ARTD9/PARP9, ARTD8/PARP14, E3 ubiquitin ligase, DTX3L/BBAP, Proliferation, Survival, Migration, STAT1, STAT3

## Abstract

**Background:**

Prostate cancer (PCa) is one of the leading causes of cancer-related mortality and morbidity in the aging male population and represents the most frequently diagnosed malignancy in men around the world. The Deltex (DTX)-3-like E3 ubiquitin ligase (DTX3L), also known as B-lymphoma and BAL-associated protein (BBAP), was originally identified as a binding partner of the diphtheria-toxin-like macrodomain containing ADP-ribosyltransferase-9 (ARTD9), also known as BAL1 and PARP9. We have previously demonstrated that ARTD9 acts as a novel oncogenic survival factor in high-risk, chemo-resistant, diffuse large B cell lymphoma (DLBCL). The mono-ADP-ribosyltransferase ARTD8, also known as PARP14 functions as a STAT6-specific co-regulator of IL4-mediated proliferation and survival in B cells.

**Methods:**

Co-expression of DTX3L, ARTD8, ARTD9 and STAT1 was analyzed in the metastatic PCa (mPCa) cell lines PC3, DU145, LNCaP and in the normal prostate luminal epithelial cell lines HPE and RWPE1. Effects on cell proliferation, survival and cell migration were determined in PC3, DU145 and/or LNCaP cells depleted of DTX3L, ARTD8, ARTD9, STAT1 and/or IRF1 compared to their proficient control cells, respectively. In further experiments, real-time RT-PCR, Western blot, immunofluorescence and co-immunoprecipitations were conducted to evaluate the physical and functional interactions between DTX3L, ARTD8 and ARTD9.

**Results:**

Here we could identify DTX3L, ARTD9 and ARTD8 as novel oncogenic survival factors in mPCa cells. Our studies revealed that DTX3L forms a complex with ARTD8 and mediates together with ARTD8 and ARTD9 proliferation, chemo-resistance and survival of mPCa cells. In addition, DTX3L, ARTD8 and ARTD9 form complexes with each other. Our study provides first evidence that the enzymatic activity of ARTD8 is required for survival of mPCa cells. DTX3L and ARTD9 act together as repressors of the tumor suppressor IRF1 in mPCa cells. Furthermore, the present study shows that DTX3L together with STAT1 and STAT3 is implicated in cell migration of mPCa cells.

**Conclusions:**

Our data strongly indicate that a crosstalk between STAT1, DTX3L and ARTD-like mono-ADP-ribosyltransferases mediates proliferation and survival of mPCa cells. The present study further suggests that the combined targeted inhibition of STAT1, ARTD8, ARTD9 and/or DTX3L could increase the efficacy of chemotherapy or radiation treatment in prostate and other high-risk tumor types with an increased STAT1 signaling.

## Introduction

Prostate cancer (PCa) is a clinically and molecularly heterogeneous disease that is characterized by its aggressive metastasization [1-3]. PCa is one of the leading causes of cancer-related mortality and morbidity in the aging male population and represents the most frequently diagnosed malignancy in men around the world [1,2]. Patients diagnosed with PCa and *de novo* metastatic tumors are generally treated with androgen deprivation therapy (ADT) since the growth of PCa is originally androgen-dependent [1,2]. However, ADT is primarily palliative, nearly all patients will eventually develop the androgen-independent and highly metastatic forms of PCa termed castration-resistant PCa (CRPC) [1,2]. Docetaxel-based chemotherapy remains the first-line treatment for men diagnosed with CRPC providing modest survival and palliative benefits [1,2,4]. Unfortunately, chemotherapy resistance develops in more than half of all CRPC patients and remains the major obstacle in treatment of CRPC [1,2,4]. Attempts to improve survival of cancer patients largely depend on strategies to target the tumor cell resistance. A common feature of PCa is the dependence on nuclear factor kappa B and the activated signal transducer and activators of transcription (STAT). Several studies have shown that STAT3 and STAT5 are required for cell growth, proliferation, survival, invasion and metastasis of many PCa subtypes [1,2,5-10]. In addition, STAT1 has been recently identified as a proto-oncogene product in a variety of cancers, including metastatic PCa (mPCa) [11-23]. A recent study has shown that 29% of clinical human mPCa’s analyzed, constitutively expressed STAT1 and IFN-stimulated genes *in vivo*[12]. STAT1 has been initially suggested to act exclusively as a suppressor of tumorigenesis, by activating growth-inhibitory and pro-apoptotic signaling in tumor cells, mainly mediated by interferon response factor (IRF)-1 [24-27].

IFNγ/STAT1 signaling is mediated through activation of IFNγ receptor and Janus kinases (JAK) 1 and 2 that lead to tyrosine phosphorylation of STAT1 on Y701, homodimerization and translocation of STAT1 to the nucleus where it induces the transcription of IFNγ-stimulated genes, including the tumor suppressor IRF1 [28]. Phosphorylation on Y701 enhances the phosphorylation on S727 in the transactivation domain of STAT1α [29-31].

Several studies have demonstrated that chemotherapeutic agents, such as doxorubicin, docetaxel or anthracyclines enhance the expression of STAT1 and its activation in chemo-resistant cancer cells [11,12,14,32]. STAT1 has been shown to be required for the observed P-glycoprotein-independent chemo-resistance towards docetaxel [15]. Several mechanisms have been reported to mediate docetaxel resistance in metastatic CRPC, such as those mediated by the P-glycoprotein/ABC multidrug transporter family [33-35], the STAT1-AKT1-clusterin axis with its pro-survival functions [15,36] and via constitutive activation of the CXCR4, ERK1/2 and c-Myc signaling loop [37]. STAT1 has therefore been suggested as a potential target for chemo-sensitization of aggressive tumors that constitutively overexpress IFNγ/STAT1-dependent pathways [12].

We have previously demonstrated that the ADP-ribosyltransferase-9 (ARTD9), also known as B-aggressive lymphoma protein (BAL1) and PARP9, acts as a novel oncogenic survival factor in high-risk, chemo-resistant, host response (HR) sub-types of diffuse large B-Cell lymphoma (HR-DLBCL) and as a crucial negative and positive co-regulator of IFNγ/STAT1-signaling [23]. ARTD9 is an inactive mono-ADP-ribosyltransferase belonging to the intracellular Diphteria toxin-like glutamate/aspartate-specific mono- and polymerizing-ADP-ribosyltransferase (ARTD) family (also known as PARPs) [38] that also includes the active mono-ADP-ribosyltransferase ARTD8 (also known as PARP14) [38-41]. Like ARTD9, ARTD8 contains several evolutionary conserved macrodomains, which have been recently shown to act as binding modules for free and protein-linked mono- or poly-ADP-ribose [42-44]. ARTD9 counteracts the IFNγ-dependent anti-proliferative and pro-apoptotic IFNγ-STAT1-IRF1-p53 axis and induces an oncogenic switch in high-risk HR-DLBCL that transforms STAT1 from a tumor suppressor to a proto-oncogene [23]. As a consequence, ARTD9 mediates proliferation, survival and chemo-resistance in HR-DLBCL [23]. ARTD8 has been shown to mediate survival in c-Myc-driven Burkitt lymphoma-like tumors *in vivo* and in multiple myeloma *in vitro*[39,45,46]. ARTD8 functions as a STAT6-specific co-regulator of IL4-mediated gene expression and is suggested to be involved in mediating IL4-induced proliferation and protection of B cells against apoptosis following irradiation or growth factor withdrawal [39-41].

The Deltex (DTX)-3-like E3 ubiquitin ligase (DTX3L), also known as B-lymphoma and BAL-associated protein (BBAP), was originally identified as a binding partner of ARTD9 [47-49]. DTX3L is overexpressed in subtypes of high-risk chemotherapy-resistant aggressive HR-DLBCL with an active host inflammatory response and tightly associated with intrinsic IFNγ signaling and constitutive activity of STAT1 [23,47,48]. Recent studies have provided first evidence that DTX3L and ARTD9 are also overexpressed in a variety of solid cancers, such as Ewing tumor or cervical carcinomas [46,48-52]. The *DTX3L* and *ARTD9* genes are located in a head-to-head orientation on chromosome 3q21 and share the same bidirectional IFNγ-responsive promoter [48]. DTX3L monoubiquitinates histone H4 lysine 91 and has been suggested to protect cells exposed to DNA damaging agents [53]. Targeted inhibition of DTX3L has been therefore suggested to increase the efficacy of DNA-damaging chemotherapeutic agents or radiation treatment [53]. However, the role of DTX3L in PCa, especially in the context of STAT1-signaling, has not been investigated.

In this study we identify DTX3L, ARTD8 and ARTD9 as novel oncogenic survival factors in androgen-independent CRPC-like mPCa cells. We demonstrate that DTX3L mediates together with ARTD8 and ARTD9 proliferation, chemo-resistance and survival in mPCa cells, indicating a functional and physical crosstalk between DTX3L and macrodomain-containing mono-ADP-ribosyltransferases in mPCa.

## Results and discussion

### DTX3L, ARTD8 and ARTD9 are constitutively overexpressed in mPCa associated with increased IFNγ/STAT1-signaling

**Figure 1:**
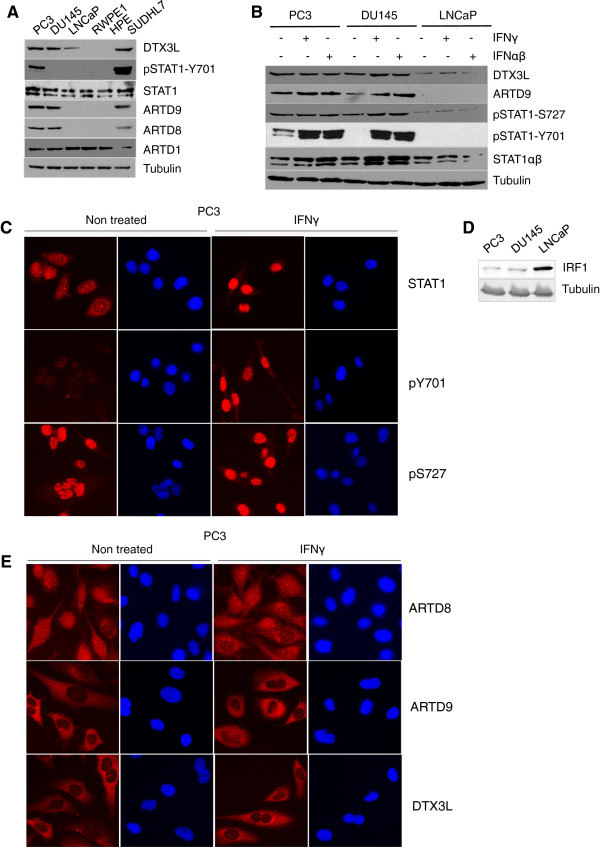
**DTX3L is constitutively overexpressed together with ARTD8 and ARTD9 in mPCa associated with increased IFNγ/STAT1 signaling. (A)** Immunoblot analyses of untreated p53-negative, mPCa cell lines PC3 and DU145, androgen-sensitive and JAK1-negative LNCaP cell line and of the immortalized normal prostate luminal epithelial cell lines HPE and RWPE1. The HR-DLBCL-SUDHL7 cell line constitutively expressing DTX3L, ARTD9 and ARTD8 [23] was used as a positive control. Whole cell extracts were separated by SDS PAGE, blotted and subsequently probed with antibodies for DTX3L, ARTD1, ARTD8 and ARTD9 pSTAT1(Y701) and tubulin. **(B)** Immunoblot analyses of STAT1-signaling in p53-negative mPCa cell lines PC3 and DU145 and in the androgen-sensitive and JAK1-negative LNCaP cell line treated with or without IFNγ or IFNαβ. PC3, DU145 and LNCaP cells were treated with or without IFNγ (200 U/ml) or IFNαβ (50 U/ml each) for 2 h and then whole cell extracts separated by SDS PAGE and subsequently probed with antibodies for DTX3L, ARTD9, STAT1, pSTAT1(Y701), pSTAT1(S727) and tubulin. The immunoblots are representative of at least three independent experiments. **(C)** Immunofluorescence microscopy analyses and sub-cellular localization of endogenous STAT1, pSTAT1-(pY701) and pSTAT1-(pS727) in PC3 cells, in presence or absence of 1000 U/ml IFNγ. Original magnification × 400. Images are representative of at least three independent experiments. **(D)** Immunoblot analyses of basal expression levels of IRF1 in PC3, DU145 and LNCaP cell lines. Whole cell extracts were separated by SDS PAGE and subsequently probed with antibodies for IRF1 and tubulin. The immunoblot is representative of at least three independent experiments. **(E)** Immunofluorescence microscopy analyses and sub-cellular localization of endogenous DTX3L, ARTD8 and ARDT9 in PC3 cells, in presence or absence of 1000 U/ml IFNγ. Original magnification × 400. Images are representative of at least three independent experiments.

The *DTX3L* gene and all three genes encoding macrodomain containing ARTD proteins (ARTD7-9) are located in the same evolutionary conserved gene cluster [48]. In order to investigate whether constitutive overexpression of DTX3L, ARTD8 and ARTD9 is associated with mPCa we analyzed their expression levels in the PCa cell lines PC3, DU145 and LNCaP [54-59], and in the normal prostate luminal epithelial cell lines HPE and RWPE1. PC3 and DU145 cells are androgen-refractory mPCa cell lines and are commonly used as CRPC models [60-62]. PC3 and DU145 cells have a high and moderate tumorigenic potential, respectively [58-62], and are highly invasive compared to the poorly tumorigenic LNCaP cells [62-65]. Contrary to the LNCaP cells, the PC3 and DU145 cells have been previously described to display enhanced basal levels of STAT1 activity and to express high levels of IL6 [15,66]. Both, PC3 and DU145 cells have been recently described to have an autocrine IL6 loop while LNCaP cells do not have any detectable IL6 secretion [66]. Our immunoblot analysis of DTX3L, ARTD8 and ARTD9, STAT1 and pSTAT1, revealed that DTX3L, ARTD8 and ARTD9 are constitutively overexpressed in the mPCa cell lines PC3 and DU145 but not in the JAK1-negative [67,68] LNCaP cells or in HPE and RWPE1 cells (Figure 1A).

We have previously demonstrated that overexpression of DTX3L and ARTD9 is tightly associated with intrinsic IFNγ-signaling and constitutively active STAT1 in HR-DLBCL [23]. We therefore examined whether constitutive overexpression of endogenous DTX3L, ARTD9 and ARTD8 is associated with STAT1 or alternatively with another STAT signaling pathway in mPCa cells. Our immunoblot analysis of DTX3L, ARTD8, ARTD9, STAT1, pSTAT1, STAT2, pSTAT2, STAT3α, STAT3αβ, pSTAT3α, STAT5, pSTAT5, STAT6 and pSTAT6 expression revealed that constitutive overexpression of DTX3L, ARTD8 and ARTD9 is indeed associated with enhanced STAT1 (pSTAT1-S727)-signaling and an autocrine IL6 loop [66] (Figure 1B-D and Additional file 1: Figure S1A-C). ARTD8 and ARTD9 were absent in LNCaP cells (Figure 1A, B and Additional file 1: Figure S1A, C). Subsequent experiments revealed that the expression of both DTX3L and ARTD9 but not of ARTD8 is dependent on JAK1 (Additional file 1: Figure S1D). A recent study has provided first evidence that expression of ARTD9 and DTX3L is induced by IL6 and strongly associated with an autocrine IL6-signaling loop in mPCa cells [66]. IL6 mainly activates STAT3, however under certain conditions, STAT1 can also be activated by IL6, [69-72]. Subsequent control experiments revealed that depletion of STAT3 in PC3 cells inhibits the expression of ARTD9 and DTX3L (Additional file 1: Figure S1E). Thus, constitutive overexpression of DTX3L and ARTD9 is likely mediated through an IL6/JAK1-STAT1:STAT3-signaling pathway in PC3 and DU145 cells in the absence of IFNγ, while further up-regulated through an IFNγ/JAK1-STAT1:STAT1-mediated signaling pathway. DTX3L was still expressed in LNCaP cells, though to a much lesser extend (Figure 1A, B Additional file 1: Figure S1A), suggesting that DTX3L can be regulated in a cell type-specific manner, independently of ARTD9, IFNγ/STAT1 and IL6/STAT3 signaling in mPCa cells.

Both, PC3 and DU145 cells showed high basal levels of transcriptionally active pSTAT1α(pS727) in the nucleus (Figure 1B, C, Additional file 1: Figure S1B and Additional file 2: Figure S2A), while PC3 cells showed enhanced basal levels of activated STAT1α/β(pY701) (Figure 1A-C and Additional file 1: Figure S1B, C). The JAK1-negative LNCaP cell line only shows low basal levels of transcriptionally active pSTAT1α(pS727) but did not show any enhanced basal levels of activated STAT1α/β(pY701) (Figure 1B and Additional file 2: Figure S2B). Phosphorylation on S727 in the transactivation domain of STAT1α can also occur independently of STAT1 tyrosine phosphorylation [73], indicating that heterodimerization with other (constitutively) tyrosine phosphorylated STATs such as STAT3 may be required for nuclear translocation of STAT1 in absence of phosphorylation on Y701 [69,74]. However, basal levels of constitutively active STAT1 in PC3 and DU145 cells are not comparable with those previously observed in the P-glycoprotein independent STAT1-AKT1-clusterin mediated docetaxel-resistant residual cell lines PC3-DR and DU145-DR [15,33,36,75-77]. The basal levels of active STAT1 (pSTAT1α-S727 and pSTAT1α/β-Y701) observed in PC3-DR or DU145-DR are highly similar to those previously observed in chemo-resistant HR-DLBCL cell lines such as SUDHL7 [23].

We have previously demonstrated that ARTD9 inhibits the transcriptional activation of tumor suppressor IRF1 in HR-DLBCL [23]. We therefore tested whether the expression of IRF1 is negatively correlated with the expression of DTX3L and ARTD9 in mPCa. Indeed, the tumor suppressor IRF1 is constitutively up-regulated in absence of DTX3L and ARTD9 in LNCaP cells, while down-regulated in presence of DTX3L and ARTD9 in PC3 and DU145 cells (Figure 1D and Additional file 2: Figure S2C). These observations suggest that DTX3L and ARTD9 might act together as transcriptional repressors of the *IRF1* gene in mPCa cells.

In agreement with previous studies in HR-DLBCL [23,48], DTX3L and ARTD9 were mainly localized in the cytoplasm whereas only small subfractions show nuclear localization (Figure 1E). Conversely, ARTD8 was evenly distributed in the nucleus and cytoplasm in these cells (Figure 1E). DTX3L is a nucleocytoplasmic shuttling protein and complex formation between DTX3L and ARTD9 in the nucleus has been suggested to facilitate the nuclear export of ARTD9 by DTX3L [48]. However, our subsequent siRNA-knockdown experiments revealed that endogenous DTX3L does not facilitate the nuclear export of ARTD9 in PC3 cells. ARTD9 was mainly localized in the cytoplasm in both PC3-siMock and PC3-siDTX3L cells (Additional file 3: Figure S3A, B and Additional file 4: Figure S4A, B). The same pattern was observed for DTX3L in PC3-siMock and PC3-siARTD9 cells (Additional file 3: Figure S3A, C and Additional file 4: Figure S4A, C), strongly indicating that the nuclear shuttling of ARTD9 is mainly regulated by other factors, and thus, the previously observed nuclear export of ectopically overexpressed fluorescent protein-tagged-ARTD9 by ectopically overexpressed fluorescent protein-tagged-DTX3L [48] most likely represents a mechanism highly specific to the cell type and stimuli.

### Crosstalk among DTX3L, ARTD8 and ARTD9 mediates proliferation in mPCa cells

**Figure 2:**
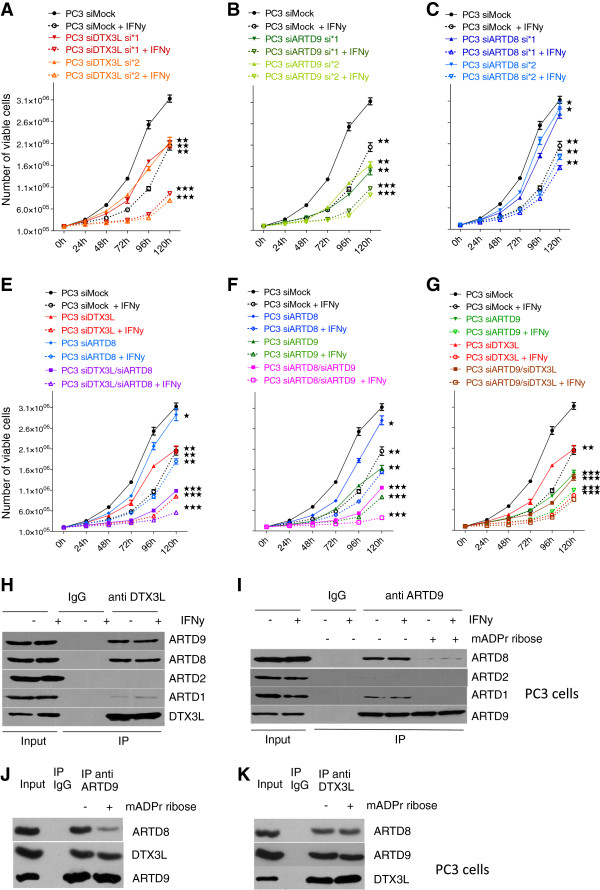
**Crosstalk among DTX3L, ARTD8 and ARTD9 mediates proliferation in PC3 cells. (A-F)** Cell proliferation analyses of PC3-siMock, PC3-siDTX3L, PC3-siARTD8, PC3-siARTD9 single knockdown cells **(A-C)** and PC3-siDTX3L/siARTD8, PC3 siDTX3L/siARTD9 or PC3-siARTD9/siARTD8 double knockdown cells **(D-F)** in presence or absence of IFNγ (200 U/ml) was assessed by the trypan blue exclusion assay. Relative cell proliferation and cell numbers are presented as mean from three independent experiments performed in triplicates. All error bars represent the SE. Statistical analysis was performed using the Student's t test. *P < 0.05, **P < 0.001 and ***P < 0.0001. **(G)** Co-immunoprecipitation analyses of endogenous DTX3L and ARTD family members in PC3 cells. PC3 cells were stimulated for 1 h with or without IFNγ (200 U/ml) and endogenous DTX3L complexes were then co-immunoprecipitated, separated on SDS PAGE, blotted and subsequently probed with antibodies for DTX3L, ARTD1 (positive control), ARTD2, ARTD8 and ARTD9. **(H)** Interaction of endogenous ARDT9 and ARTD1 or ARTD8 is mediated by (mono)-ADP-ribosylation. PC3 cells were stimulated for 1 h with IFNγ (200 U/ml) and endogenous ARTD9-ARTDx complexes subsequently co-immunoprecipitated in presence or absence of 5 mM mono-ADP-ribose using an anti-ARTD9 antibody. Complexes were then separated on SDS PAGE, blotted and subsequently probed with antibodies against endogenous ARTD1 (positive control), ARTD2, ARTD8 and ARTD9. **(I, J)** Interaction between endogenous DTX3L and ARTD8 or ARTD9 is independent of (mono)-ADP-ribosylation. Endogenous DTX3L-ARTD8/9 complexes were co-immunoprecipitated from extracts of PC3 cells in presence or absence of 5 mM mono-ADP-ribose using either an anti-ARTD9 **(I)** or an anti-DTX3L **(J)** antibody. Complexes were then separated on SDS PAGE, blotted and subsequently probed with antibodies against endogenous ARTD8, ARTD9 and DTX3L.

In order to investigate whether a crosstalk among DTX3L, ARTD8 and ARTD9 mediates proliferation in mPCa cells we first analyzed the proliferation of cells depleted of DTX3L, ARTD8 or ARTD9 using siRNA (Additional file 5: Figure S5A, B) in presence or absence of IFNγ. IFNγ is known to inhibit the proliferation of p53-negative, androgen-refractory mPCa cells [78,79]. These experiments revealed that knockdown of DTX3L or ARTD9 in PC3 cells strongly inhibits proliferation when compared to control cells (Figure 2A, B), whereas knockdown of ARTD8 only showed a minor effect on proliferation (Figure 2C). As expected, knockdown of DTX3L or ARTD9 in LNCaP cells did not affect cell growth in LNCaP cells (data not shown). We next analyzed the effect on proliferation upon double knockdown of ARTD8/DTX3L, ARTD9/DTX3L or ARTD8/ARTD9 in PC3 cells. These analyses revealed that ARTD8 acts synergistically or additively together with DTX3L and ARTD9 in proliferation, strongly suggesting a functional crosstalk between ARTD8, DTX3L and ARTD9 (Figure 2D, E). No significant additional effects on proliferation were observed in siDTX3L/siARTD9 double knockdown cells indicating that DTX3L and ARTD9 regulate the same signaling pathway(s) in proliferation (Figure 2F).

DTX3L was originally identified as an ADP-ribosylation independent binding partner of ARTD9, interacting with the catalytic domain of ARTD9 [47]. Moreover, a recent study suggested that DTX3L interacts through ARTD9 with ARTD1 (also known as PARP1) in a DNA damage and poly-ADP-ribosylation-dependent manner [80]. We therefore investigated whether DTX3L forms endogenous complexes with ARTD8 under normal physiological conditions. Indeed, our co-immunoprecipitation studies in PC3 cells revealed that endogenous DTX3L forms strong complexes with ARTD8 and ARTD9 (Figure 2G, J and Additional file 5: Figure S5C, D). However, endogenous DTX3L barely interacted with ARTD1 under normal physiological conditions (Figure 2G). No interaction was observed with ARTD2 (also known as PARP2) (Figure 2G). Subsequent co-immunoprecipitation experiments with endogenous ARTD8, ARTD9 and other ARTDs in PC3 cells revealed that endogenous ARTD9 strongly interacts with ARTD8 (Figure 2H, I and Additional file 5: Figure S5D, F) and also interacts to a lesser extent with other active mono-ADP-ribosyltransferases (Additional file 5: Figure S5E). ARTD9 only interacted weakly with ARTD1 (Figure 2H), whereas no interaction was observed with ARTD2 (Figure 2H). These experiments revealed that the observed interactions between ARTD9 and active mono-ADP-ribosyltransferases are mediated by (mono)-ADP-ribosylation (Figure 2H, I and Additional file 5: Figure S5D, E) and thus very likely mediated through their macrodomains. Several studies have demonstrated that the interaction between macrodomain-containing ARTDs and (mono)-ADP-ribosylated proteins, including active mono-ARTD enzymes, such as ARTD8 and ARTD10 (also known as PARP10), is mediated through their macrodomains [44,80,81]. Conversely, the observed interaction between DTX3L and ARTD8 or ARTD9 is not dependent on ADP-ribosylation ( [47], Figure 2J and Additional file 5: Figure S5D), indicating that DTX3L could form different (mono)-ADP-ribosylation dependent and (mono)-ADP-ribosylation independent complexes with ARTD8 and ARTD9. Given that ARTD8 does not function as a coactivator for STAT1 [40] it is very likely that different DTX3L-ARTDx complexes simultaneously exist and do act in distinct signaling pathways.

### Crosstalk among DTX3L, ARTD8 and ARTD9 mediates chemo-resistance and survival in mPCa cells in an ADP-ribosylation-dependent manner

**Figure 3:**
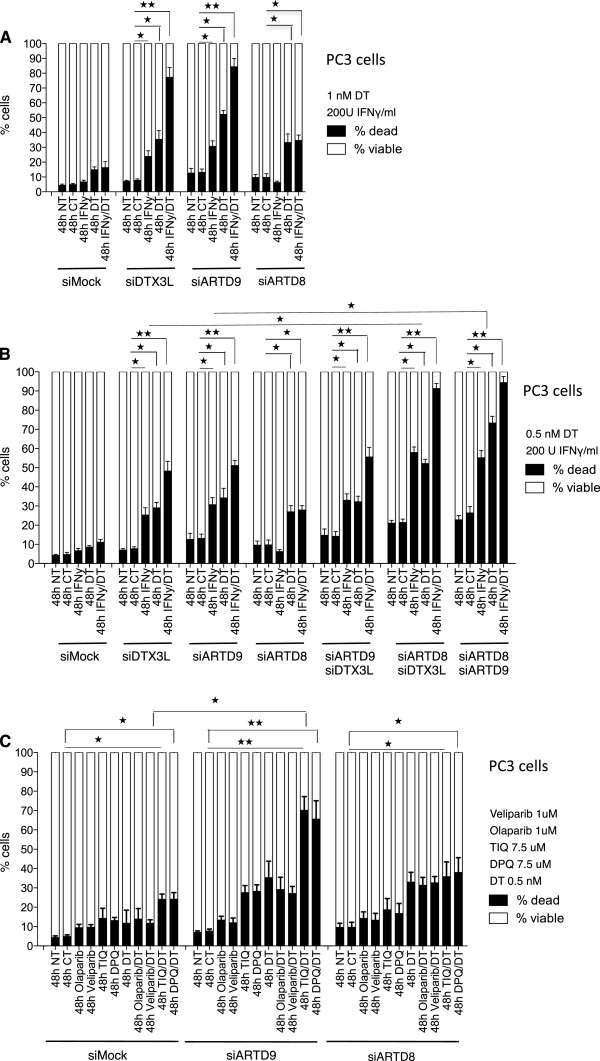
**Crosstalk among DTX3L, ARTD8 and ARTD9 mediates chemo-resistance and survival in PC3 cells and is dependent on ADP-ribosylation. (A)** Cell viability analyses of PC3-siMock, PC3-siDTX3L, PC3-siARTD8 and PC3-siARTD9 single knockdown cells were assessed by the trypan blue exclusion assay. Cells were treated as indicated with IFNγ and/or docetaxel (DT) and counted after 48 h, NT: not treated, CT: control treatment (solvent). Values represent the means of three independent experiments, and the error bars represent the SE. Statistical analysis was performed using the Student's t test. *P < 0.05, **P < 0.001 and ***P < 0.0001 **(B)** Cell viability analyses of PC3-siMock, PC3-siDTX3L/siARTD8, PC3-siDTX3L/siARTD9 or PC3-si ARTD9/siARTD8 double knockdown cells were assessed by the trypan blue exclusion assay. Cells were treated as indicated with IFNγ and/or docetaxel (DT) and counted after 48 h, NT: not treated, CT: control treatment (solvent). Values represent the means of three independent experiments, and the error bars represent the SE. Statistical analysis was performed using the Student's t test. *P < 0.05, **P < 0.001 and ***P < 0.0001. **(C)** Cell viability analyses of PC3-siMock, PC3-siDTX3L**,** PC3-siARTD8 and PC3-siARTD9 single knockdown cells treated in presence or absence of docetaxel (DT) (0.5 nM) with the ARTD1/2-specific inhibitors Olaparib (1 μM) and Veliparib (1 μM) or with the more ARTD7/8-specific inhibitors DPQ (7.5 μM) and TIQ-A (7.5 μM) were assessed by the trypan blue exclusion assay. Values represent the means of three independent experiments performed in triplicate, and the error bars represent the SE. Statistical analysis was performed using the Student's t test. *P < 0.05, **P < 0.001 and ***P < 0.0001.

In order to strengthen our previous observation in DLBCL [23], we subsequently analyzed the chemo-resistance and survival in PC3 cells depleted of either DTX3L, ARTD8 or ARTD9 in the presence or absence of IFNγ and/or docetaxel. Indeed we found that DTX3L, ARTD8 and ARTD9 mediate survival and chemo-resistance in mPCa cells (Figure 3A and Additional file 6: Figure S6A-F). To address whether there is a functional crosstalk between DTX3L, ARTD8 and ARTD9 in mediating survival and chemo-resistance, we performed double knockdown studies in PC3 cells. Interestingly, upon simultaneous depletion of ARTD8/DTX3L, ARTD9/DTX3L and ARTD8/ARTD9, IFNγ could enhance the sensitivity towards docetaxel in the absence of DTX3L or ARTD9. These results strongly suggest that both DTX3L and ARTD9, but not ARTD8, counteract the IFNγ-dependent anti-proliferative and pro-apoptotic IFNγ-STAT1-IRF1 axis [23] (Figure 3B). No significant additional effects on survival and chemo-resistance were observed in siDTX3L/siARTD9 double knockdown cells indicating that DTX3L and ARTD9 regulate the same signaling pathways in survival and chemo-resistance (Figure 3B). Together, these results indicate that there is a functional crosstalk between DTX3L, ARTD8 and ARTD9 in survival and chemo-resistance in mPCa cells. ARTD8 does not influence STAT1 signaling directly but through other mechanisms. A recent study in mice has provided first evidence that ARTD8 functions as a STAT6-specific co-regulator of IL4-mediated gene expression and is involved in IL4-induced proliferation and protection of B cells against apoptosis following irradiation or growth factor withdrawal [39]. Although no clear correlation between STAT6 expression or activity and ARTD8 could be observed in mPCa cell lines, it is very likely that ARTD8 might act together with DTX3L as a STAT6-specific survival factor in mPCa cells. STAT6 has been recently shown to act as a survival factor and to enhance mPCa progression [82]. Alternatively, ARTD8 might act together with DTX3L independently of STAT6 signaling in these cell lines.

Our finding of a (mono)-ADP-ribosylation-dependent interaction between ARTD8 and ARTD9 strongly suggests that the enzymatic activity of mono-ADP-ribosyltransferases is required for this interaction. Thus, we analyzed the effects of the enzymatic activity of ARTD8 or other ARTDs on survival and proliferation of mPCa cells. A recent study suggested that ARTD9 and ARTD1 physically and functionally interact and together mediate survival in response to genotoxic stress [80]. In order to test this hypothesis we treated ARTD8- or ARTD9-depleted PC3 cells in presence or absence of docetaxel with the ARTD1/2-specific inhibitors Olaparib and Veliparib [83-86] or with the more ARTD7/8-specific inhibitors DPQ and TIQ-A [83-85]. ARTD8- or ARTD9-depleted cells treated with Olaparib and Veliparib only showed a minor increase in cell death when compared to control cells. (Figure 3C). Remarkably, treatment with DPQ and TIQ-A strongly increased cell death in ARTD9-depleted cells when compared to control cells (Figure 3C). Conversely, in ARTD8-depleted cells we did not observe an increase in cell death upon DPQ and TIQ-A treatment when compared to control cells (Figure 3C), indicating that one of the definitive targets of DPQ and TIQ-A is the enzymatic activity of ARTD8. Moreover, we did not observe any functional crosstalk between ARTD1 and ARTD8 or ARTD9 n PC3 cells under the tested conditions (Figure 3C). In line with these observations, overexpression of active ARTD8 wild type in PC3 cells enhanced survival in siMock cells and rescued the effects on cell survival in siARTD8 knockdown cells. In contrast, overexpression of a catalytically inactive ARTD8 mutant form in ARTD8-depleted PC3 cells did not increase cell survival in siMock cells or siARTD8 knockdown cells (Additional file 6: Figure S6G). These results strongly suggest that the enzymatic activity of ARTD8 is required for the survival of mPCa cells.

### DTX3L and ARTD9 mediate proliferation, chemo-resistance and survival in mPCa cells in a STAT1-dependent manner

**Figure 4:**
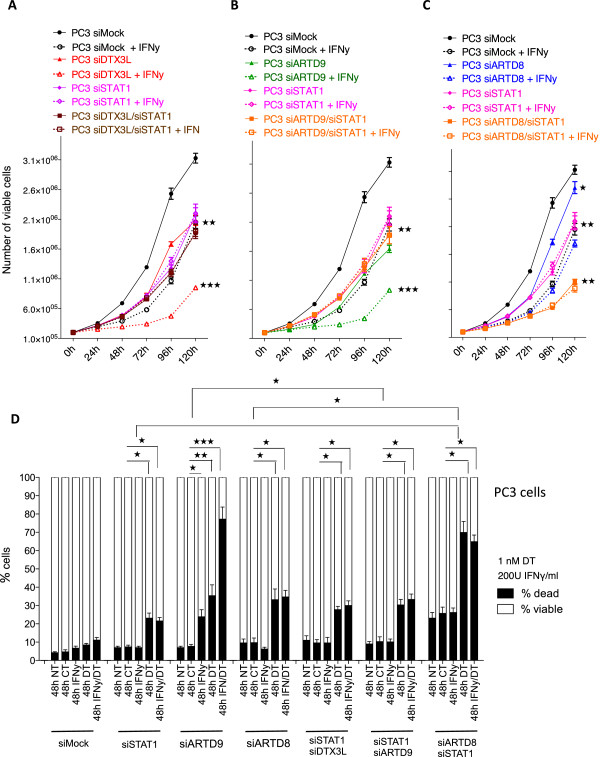
**DTX3L- and ARTD9-mediated proliferation, chemo-resistance and survival in PC3 cells is dependent on STAT1. (A-C)** Cell proliferation analyses of PC3-siMock, PC3-siSTAT1, PC3-siSTAT1/siDTX3L **(A)**, PC3-siSTAT1/siARTD8 **(B)**, or PC3-siSTAT1/siARTD9 **(C)**, double knockdown cells in presence or absence of IFNγ (200 U/ml) were assessed by the trypan blue exclusion assay. Relative cell proliferation and cell numbers are presented as mean from three independent experiments performed in triplicate, the error bars represent the SE. Statistical analysis was performed using the Student's t test. *P < 0.05, **P < 0.001 and ***P < 0.0001. **(D)** Cell viability analyses of a PC3-siMock, PC3-siSTAT1 single knockdown, and PC3-siSTAT1/siDTX3L PC3-siSTAT1/siARTD8 or PC3-siSTAT1/siARTD9 double knockdown cells were assessed by the trypan blue exclusion assay. Cells were treated as indicated with IFNγ and/or docetaxel (DT), NT: not treated, CT: control treatment (solvent). Values represent the means of three independent experiments performed in triplicates, and the error bars represent the SE. Statistical analysis was performed using the Student's t test. *P < 0.05, **P < 0.001 and ***P < 0.0001.

Several studies strongly suggest that STAT1α activates anti-proliferative and pro-apoptotic genes (i.e. mediated through the IFNγ-STAT1-IRF1-p53 axis) while concomitantly activating anti-apoptotic-pro-survival pathways (i.e. mediated through the STAT1-IRF2/BCL2-axes) [23,87,88]. In addition, overexpression of STAT1β, the antagonistic isoform of STAT1α, increases the growth rate of cells and their resistance to drug-induced apoptosis and cell cycle arrest by repressing STAT1α target genes such as p21 and IRF1 [87]. Our previous study has demonstrated that ARTD9-mediated cell proliferation, chemo-resistance and cell survival in HR-DLBCL is dependent on STAT1 [23].In order to examine whether depletion of STAT1 might inhibit the pro-apoptotic and/or anti-proliferative IFNγ-STAT1-IRF1-axis in absence of DTX3L or ARTD9 we next analyzed cell proliferation (Figure 4A-C) and cell survival (Figure 4D) defects in PC3 cells simultaneously depleted of DTX3L/STAT1, ARTD8/STAT1 and ARTD9/STAT1. Indeed, depletion of STAT1 did not further inhibit cell proliferation, chemo-resistance and cell survival in the absence of ARTD9 or DTX3L, when compared to the single knockdown cells (Figure 4A, B, D). However, the observed proliferation defects and the increase in cell death upon depletion of DTX3L or ARTD9 could not be fully rescued by simultaneous depletion of DTX3L/STAT1 and ARTD9/STAT1, when compared to single knockdown and control cells (Figure 4A-D). However, depletion of STAT1 alone strongly affected cell proliferation (Figure 4A, B), chemo-resistance and cell survival (Figure 4D), indicating that STAT1 itself is required for cell proliferation, chemo-resistance and cell survival. Conversely, cell proliferation and survival of ARTD8-depleted cells is even more inhibited upon additional depletion of STAT1 (Figure 4C, D), clearly indicating that STAT1 and ARTD8 do not act together in the same signaling pathway. Given that depletion of STAT1 alone strongly affected cell proliferation and survival, but did not further inhibit cell proliferation and survival in the absence of ARTD9 or DTX3L (Figure 4A, B), indicates that DTX3L and ARTD9 together with STAT1 act in the same signaling pathways.Taken together these results suggest that both DTX3L and ARTD9 mediate cell proliferation, survival and chemo-resistance of mPCa cells in a STAT1-dependent manner whereas ARTD8 enhances survival and chemo-resistance independently of STAT1 (Figure 4A-D).

### DTX3L and ARTD9 repress expression of the tumor suppressor IRF1 in PCa cells

**Figure 5:**
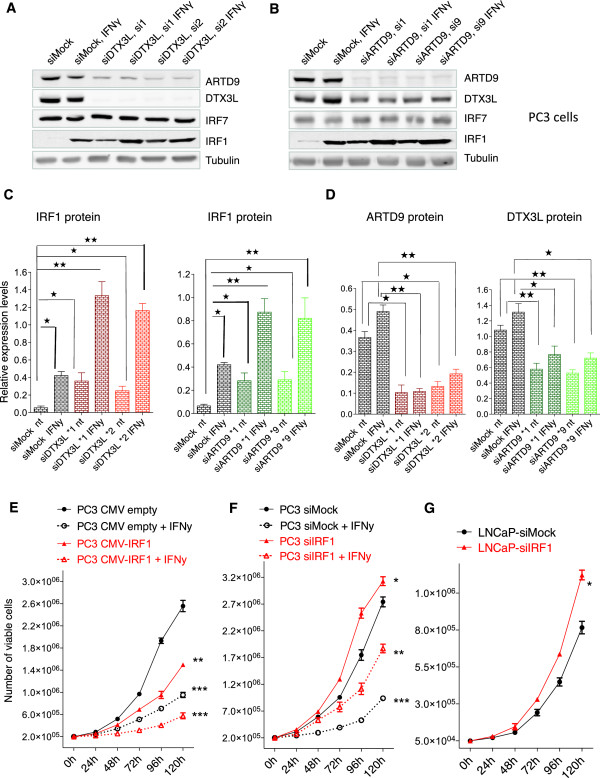
**DTX3L and ARTD9 repress tumor suppressor IRF1 expression in mPCa cells. (A and B)** Immunoblot analyses of the tumor suppressor gene product IRF1. PC3-siMock, PC3-siDTX3L **(A)** or PC3-siARTD9 **(B)** single knockdown cells were treated with or without IFNγ (200 U/ml) for 6 h and then whole cell extracts separated by SDS PAGE, blotted and subsequently probed with antibodies for ARTD9, DTX3L, IRF1, IRF7 and tubulin. The immunoblots are representative of at least three independent experiments. **(C)** Quantification of IRF1 levels shown in Figure 5A, B. IRF1 levels were normalized to tubulin. Values represent the means of three independent experiments. **(D)** Quantification of ARTD9 and DTX3L protein levels in PC3-siMock, PC3-siDTX3L and PC3-siARTD9 single knockdown, respectively, as represented in Figure 5A, B. ARTD9 and DTX3L protein levels were normalized to tubulin. Values represent the means of three independent experiments. **(E)** Cell proliferation analyses of PC3-CMVprom-empty-control and PC3-CMVprom-IRF1 cells were assessed in presence or absence of IFNγ (100U/ml) by the trypan blue exclusion assay. Relative cell proliferation and cell numbers are presented as means of three independent experiments performed in triplicates. **(F)** Cell proliferation analyses of PC3-siMock, PC3-siIRF1 and PC3-siARTD9 single knockdown cells were assessed in presence or absence of IFNγ (200 U/ml) by the trypan blue exclusion assay. Relative cell proliferation and cell numbers are presented as means of three independent experiments performed in triplicate. **(G)** Cell proliferation analyses of LNCaP-siMock and LNCaP-siIRF1 single knockdown cells were assessed by the trypan blue exclusion assay. Relative cell proliferation and cell numbers are presented as means of two independent experiments performed in triplicate. All error bars shown in A to G represent the SE. Statistical analysis was performed using the Student's t test. *P < 0.05, **P < 0.001 and ***P < 0.0001.

ARTD9 can bind to the *IRF1*-promoter and together with STAT1β inhibits the transcription of the *IRF1* gene, thereby counteracting the IFNγ-dependent anti-proliferative and pro-apoptotic IFNγ-STAT1-IRF1-p53 axis in high-risk HR-DLBCL [23]. We therefore investigated whether DTX3L and/or ARTD9 function as transcriptional repressors of IRF1 and regulate STAT1 signaling in PCa cells. Our current study demonstrates that DTX3L and ARTD9 act together as repressors of the tumor suppressor IRF1 in mPCa. The basal IRF1 protein and mRNA expression levels were strongly increased in PC3 cells depleted of DTX3L or ARTD9 in absence of IFNγ (Figure 5A-C and Additional file 7: Figure S7C). The IRF1 protein and mRNA expression was further increased in PC3 cells depleted of DTX3L or ARTD9 upon IFNγ treatment (Figure 5A-C and Additional file 7: Figure S7C). Subsequent *IRF1*-promoter-driven luciferase reporter assays in PC3 cells showed that overexpression of DTX3L or ARTD9 together with STAT1β down-regulates the *IRF1*-promoter-driven luciferase reporter (Additional file 7: Figure S7D-F). Moreover, co-overexpression of all three proteins together (DTX3L, ARTD9 and STAT1β) synergistically down-regulated the *IRF1*-promoter-driven luciferase reporter (Additional file 7: Figure S7G). Together, these results suggest that ARTD9 and DTX3L cooperate and act as transcriptional repressors by forming a ternary complex with STAT1β. It remains to be investigated whether DTX3L might monoubiquitinate histone H4 lysine 91 on the *IRF1*-promoter and thereby inhibits the transcription of the *IRF1* gene. In addition, our knockdown experiments demonstrate that DTX3L and ARTD9 positively regulate the expression of each other on their own gene expression level, though to a different extent (Figure 5A, B, D). Depletion of DTX3L strongly inhibited the expression of ARTD9 whereas depletion of ARTD9 inhibited the expression of DTX3L to a minor extent. We have previously shown that ARTD9 can bind to its own bidirectional promoter [23]. Thus, DTX3L and ARTD9 positively regulate each other directly through their shared bidirectional promoter.

To confirm these observations, we next analyzed the effect of IRF1 on proliferation and cell survival by either depletion or overexpression of IRF1 in PCa cells. Exogenous overexpression of human IRF1 in PC3 cells (Additional file 8: Figure S8A) revealed that the presence of IRF1 indeed strongly inhibited proliferation of PC3 cells (Figure 5E). In line with this, knockdown of IRF1 enhanced the proliferation of PC3 (Figure 5F and Additional file 8: Figure S8B, C) and of LNCaP cells (Figure 5G and Additional file 8: Figure S8D), although to a lesser extent in the JAK1-negative LNCaP cell line (Figure 5G). Several studies suggest that phosphorylation and/or acetylation of IRF1 is required for full transcriptional activity of IRF1 [89-91]. Tyrosine phosphorylation and probably also acetylation of IRF1 appears to be dependent on active IFNγ/JAK1 signaling [89,91]. Subsequent survival assays with cells depleted of IRF1 revealed that IRF1 does not inhibit survival of mPCa cells (Additional file 8: Figure S8E), strongly indicating that other STAT1-dependent target genes are involved and/or required for the DTX3L/ARTD9-mediated effects on survival of mPCa cells. Future studies will be required in order to identify the target genes involved in these processes and elucidate the exact molecular mechanisms.

### DTX3L interacts with the IFNGR complex and antagonistically regulates together with ARTD9 the phosphorylation of STAT1 on Y701 in mPCa cells

**Figure 6:**
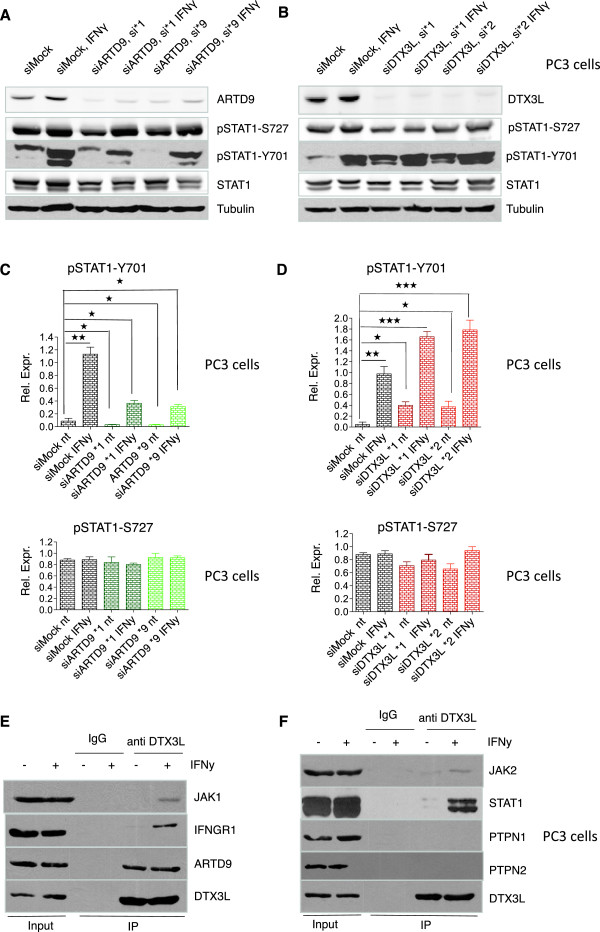
**DTX3L interacts with the IFNGR complex and together with ARTD9 antagonistically regulates the phosphorylation of STAT1 on Y701 in PC3 cells. (A and B)** Immunoblot analyses of STAT1-signaling in PC3-siMock, PC3-siDTX3L **(A)**, and PC3-siARTD9 **(B)** single knockdown cells. PC3-siMock, PC3-siDTX3L or PC3-siARTD9 single knockdown cells were treated with or without IFNγ (200 U/ml) for 2 h and then whole cell extracts separated by SDS PAGE, blotted and subsequently probed with antibodies for STAT1, pSTAT1(Y701), pSTAT1(S727) and tubulin. The immunoblots are representative of at least three independent experiments. **(C and D)** Quantification of pSTAT1(Y701) and pSTAT1(S727) levels shown in Figure 5A, B. pSTAT1(Y701) and pSTAT1(S727) levels were normalized to tubulin and STAT1. Values represent the mean of three independent experiments, and the error bar represents the SE. Statistical analysis was performed using the Student's t test. *P < 0.05, **P < 0.001 and ***P < 0.0001. **(E and F)** Co-immunoprecipitation analyses of endogenous DTX3L-IFNGR complexes in PC3 cells: Endogenous DTX3L/IFNGR complexes were co-immunoprecipitated using an anti-DTX3L antibody. Complexes were then separated on SDS PAGE, blotted and subsequently probed with antibodies against endogenous DTX3L, ARTD9, STAT1, IFNGR1, JAK1, JAK2, PTPN1 and PTPN2.

Since our previous studies showed that ARTD9 enhances phosphorylation of both isoforms of STAT1 on Y701 we tested whether DTX3L might function together with ARTD9 in regulating the phosphorylation of STAT1 on Y701 in mPCa cells. Surprisingly, these experiments revealed that DTX3L and ARTD9 antagonistically regulate the phosphorylation of both STAT1 isoforms STAT1α and STAT1β, on Y701 in mPCa cells. ARTD9 also stimulated phosphorylation of STAT1 on Y701 (Figure 6A, C) whereas DTX3L strongly inhibited phosphorylation of STAT1 on Y701 (Figure 6B, D). However, the observed effects of ARTD9 on STAT1 phosphorylation are less pronounced than previously observed in HR-DLBCL [23]. In contrast to their P-glycoprotein-independent chemo-resistant variants PC3-DR or DU145-DR [15,33,36,75-77] both PC3 and DU145 cells do not have high basal levels of constitutively tyrosine phosphorylated STAT1 (pSTAT1α(Y701) and pSTAT1β(Y701) and are sensitive towards docetaxel [15,33,36,75-77]. Thus, the siDTX3L- and siARTD9-mediated effects on STAT1-signaling are most likely much higher in the PC3-DR or DU145-DR cells [15] and are therefore more comparable with the effects observed in HR-DLBCL. Moreover, since DTX3L and ARTD9 regulate each other on their gene expression level (Figure 5A, B and D) the observed antagonistic effects are tightly balanced and might explain why the effects are not completely visible in the knockdown cells.

Our previous study has provided preliminary evidence that ARTD9 interacts with the IFNGR-receptor complex and thereby stimulates directly or indirectly the kinase activity of JAK1/2 [23]. Indeed, our co-immunoprecipitation studies revealed that endogenous DTX3L interacts with activated STAT1-containing IFNGR-receptor complexes in the cytoplasm (Figure 6E, F) and forms together with ARTD9 complexes with STAT1 in the nucleus (Additional file 8: Figure S8F). No interaction with the tyrosine phosphatases PTPN1 and PTPN2, both known to dephosphorylate pSTAT1 on Y701 [92-94], was observed (Figure 6F). Our observations strongly suggest that DTX3L and ARTD9 might antagonistically regulate the JAK1/2 kinase activity and thereby antagonistically influence the nuclear activities of both STAT1α and STAT1β. Thus, DTX3L and ARTD9 seem to be required for the fine-tuning of STAT1-signaling, particularly in tumorigenesis. Moreover, since both DTX3L and ARTD9 are target genes of STAT1 [23,48] the suggested antagonistic and cooperative activities of DTX3L and ARTD9 may represent a general negative and positive feedback loop in STAT1-signaling. Due to the fact that ARTD9 and DTX3L are regulating each other on the level of gene expression it is quite difficult to experimentally address the exact molecular mechanisms underlying the proposed antagonism between them. The observed effect on STAT1 tyrosine phosphorylation might be regulated through (mono)-ubiquitinylation and/or mono-ADP-ribosylation. We have previously shown that the interactions between ARTD9 and STAT1α/β are mediated through macrodomains and dependent on ADP-ribosylation [23]. However, we did not find any direct evidence that STATs are mono-ADP-ribosylated *in vivo*. Thus, it remains to be investigated whether (mono)-ubiquitinylation and/or mono-ADP-ribosylation is involved in this process.

### DTX3L mediates cell migration of mPCa cells in a STAT1 and/or STAT3-dependent manner

**Figure 7:**
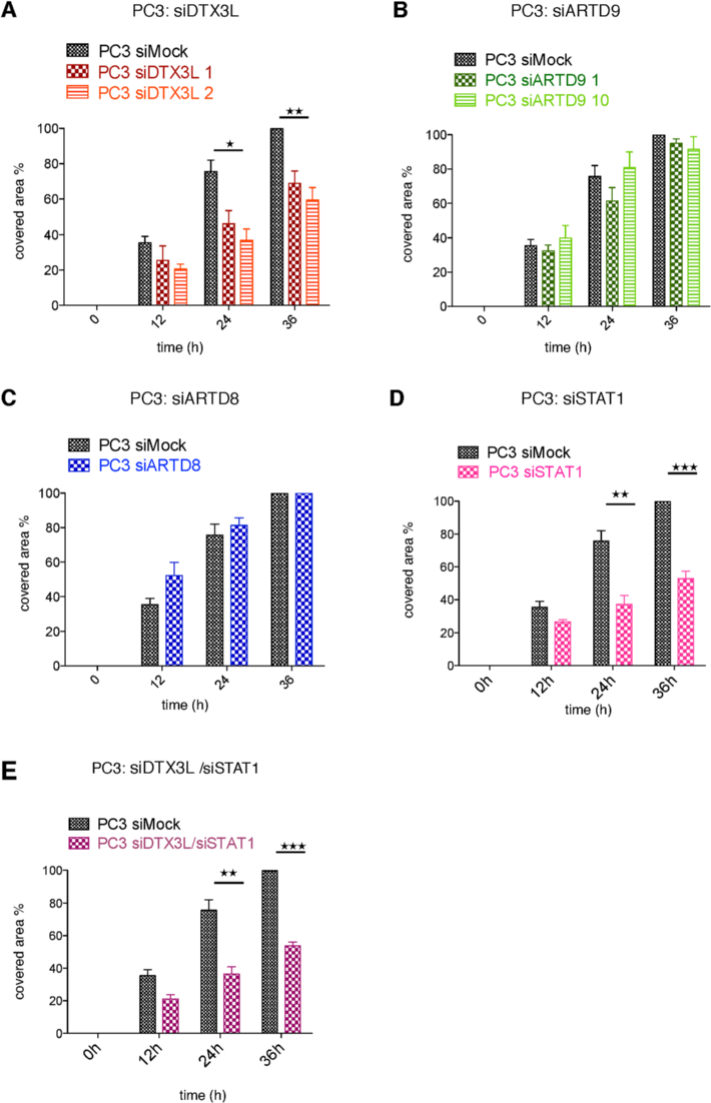
**DTX3L but not ARTD9 or ARTD8 mediates together with STAT1 cell migration of PC3 cells. (A-E)** PC3-siMock, PC3-siDTX3L **(A)**, PC3-siARTD9 **(B)**, PC3-siARTD8 **(C)**, PC3-siSTAT1 **(D)** single knockdown cells and PC3-siDTX3L/siSTAT1 **(E)** PC3-double knockdown cells were seeded into 6-well plates and treated as described in Material and Methods. At 0, 12, 24, and 36 h photographs were made and quantified as described in Material and Methods. Values represent the mean of three independent experiments and the error bars represent the SE. Statistical analysis was performed using the Student's t test. *P < 0.05, **P < 0.001 and ***P < 0.0001.

A recent study has provided first evidence that ARTD9 might be associated with lymphocyte migration and may promote the dissemination of malignant B cells in high-risk DLBCL *in vivo*[48,95]. Ectopic overexpression of ARTD9 in an ARTD9 and DTX3L-negative DLBCL cell line derived from low risk DLBCL tumor strongly enhanced the migration *in vitro* when compared to control cells [48]. In order to investigate whether endogenous DTX3L or ARTD9 are required for the migration of mPCa cells we analyzed the migration potential of PC3 cells depleted of DTX3L and ARTD9 using the classical scratch wound healing assay for adherent cell lines. Surprisingly, upon depletion of DTX3L, but not ARTD9 or ARTD8, cell migration was impaired in PC3 and DU145 cells (Figure 7A-C and Additional file 9: Figure S9A-F). These observations are in contrast to the previous study, which did not address the expression level of DTX3L in the ARTD9 ectopically overexpressing DLBCL cell line [48].

**Figure 8:**
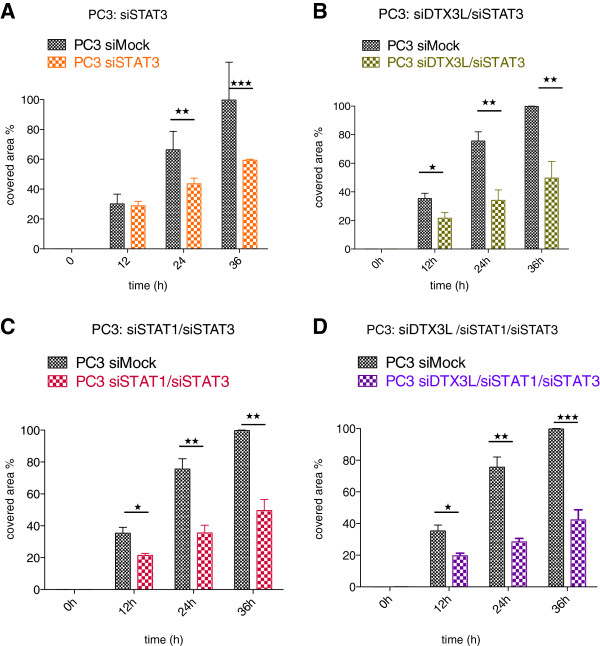
**DTX3L-mediated cell migration of PC3 cells might also be dependent on STAT3. (A-F)** PC3-siSTAT3 single knockdown cells **(A)**, PC3-siDTX3L/siSTAT3 **(B)**, PC3-siSTAT1/siSTAT3 **(C)** double knockdown cells and PC3-siDTX3L/siSTAT1/siSTAT3 **(D)** triple knockdown cells were seeded into 6-well plates and treated as described in Material and Methods. At 0, 12, 24, and 36 h photographs were made and quantified as described in Material and Methods. Values represent the mean of three independent experiments and the error bars represent the SE. Statistical analysis was performed using the Student's t test. *P < 0.05, **P < 0.001 and ***P < 0.0001.

We next investigated whether the observed effects of DTX3L on migration of mPCa cells is dependent on STAT1. Besides its role in mediating tumor survival and growth, STAT3 plays a crucial role in tumor migration, invasion and metastasis [7,69] and recent studies provided preliminary evidence that STAT1 is involved in migration of mPCa cells [20,21]. In order to evaluate whether cell migration is also dependent on STAT1 we investigated the migration potential of cells depleted of STAT1 or both STAT1 and DTX3L. These experiments showed that the observed effect is indeed dependent on STAT1-signaling (Figure 7D, E and Additional file 10: Figure S10A, B, E). Migration of PC3 and DU145 cells was not further impaired upon double knockdown of STAT1 and DTX3L when compared to single depletion of DTX3L or STAT1, strongly indicating that DTX3L and STAT1 act together in the same pathway(s) (Figure 7D, E and Additional file 10: Figure S10A, B, E). Control experiments revealed that IRF1 does not affect migration of mPCa cells **(**Additional file 10: Figure S10D), suggesting that the IFNγ/IRF1 axis is not involved in cell migration. The fact that depletion of ARTD9 and IRF1 did not affect cell migration suggests that DTX3L acts in STAT1-signaling pathways in an ARDT9/IFNγ-independent manner. The observed DTX3L-dependent effects on migration might be therefore mediated by the constitutive nuclear activity of pSTAT1α(pS727) homo- or heterodimers. Indeed, the observed impairment of cell migration upon DTX3L/STAT1 knockdown is in line with the cell migration analyzed upon depletion of STAT3 (Figure 8A and Additional file 9: Figure S9F), which was used as positive control in this set of experiments. Subsequent control experiments revealed that the observed effect might be indeed also dependent on STAT3-signaling (Figure 8B-D and Additional file 10: Figure S10F-H). No further significant inhibition was observed neither in siSTAT1/siSTAT3, siDTX3L/siSTAT3 double knockdown cells nor in siDTX3L/siSTAT1/siSTAT3 triple knockdown cells when compared to siDTX3L, siSTAT1 or siSTAT3 single knockdown cells, strongly indicating that DTX3L, STAT1 and STAT3 act in the same pathway(s) (Figure 8B-D and Additional file 10: Figure S10F-H). DTX3L might function in a non-canonical STAT1:STAT3 heterodimer-mediated signaling pathway in migration of mPCa cells.

Together, our *in vitro* cell migration analyses strongly indicate that DTX3L together with STAT1 is crucial for proliferation and survival but might also be required together with STAT1 for the metastasization and dissemination of androgen-refractory mPCa cells *in vivo*.

## Conclusions

**Figure 9:**
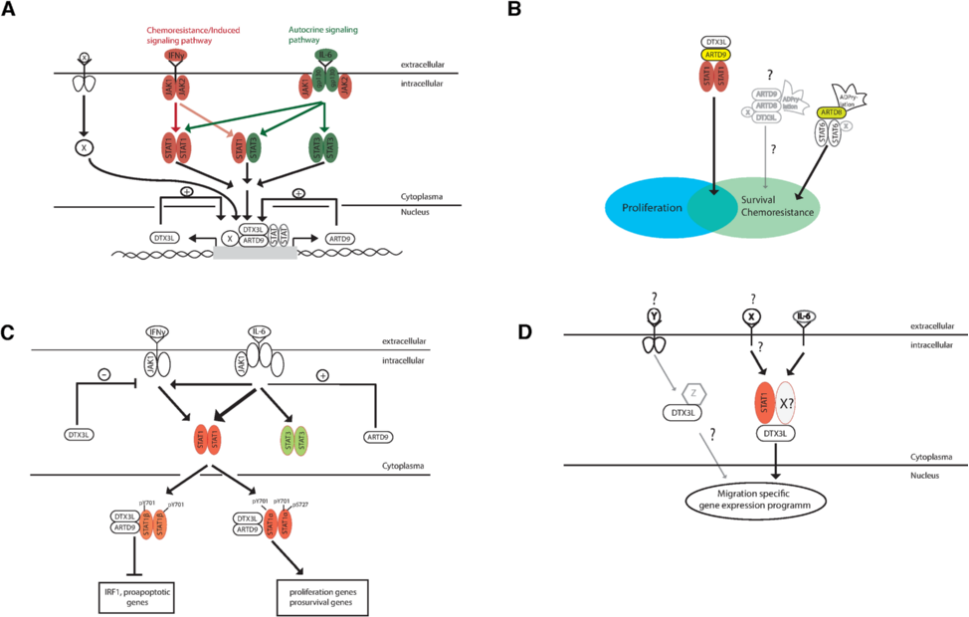
**Proposed working models for the postulated crosstalk among DTX3L, ARTD8 and ARTD9 in chemotherapy-resistant mPCa cells. (A)** Constitutively active IL6/STAT3-signaling and enhanced IFNGR-JAK1/2-STAT1- signaling in chemotherapy-resistant mPCa cells, including CRPC-like cells causes overexpression of DTX3L and ARTD9, which in turn, further stimulates their own expression through a positive feedback loop. **(B)** Crosstalk between DTX3L/ARTD9-STAT1 and ARTD8-STAT6-signaling pathways is required for proliferation and cell survival of chemotherapy-resistant mPCa cells, including CRPC-like cells. **(C)** Similar to the situation in HR-DLBCL, DTX3L and ARTD9, together with STAT1β repress the transcriptional activation of the tumor suppressor IRF1 and other anti-proliferative and pro-apoptotic genes while together with STAT1α both, DTX3L and ARTD9 might activate genes required for cell proliferation and survival of mPCa cells. **(D)** Overexpression of DTX3L but not ARTD8 or ARTD9 also mediates cell migration of mPCa cells, dependent on STAT1 and STAT3-signaling. DTX3L might form migration-specific, ARTD9-independent STAT1 homodimer or non-canonical STAT1/STAT3 heterodimer complexes.

We have identified the E3 ubiquitin ligase DTX3L and the macrodomain-containing mono-ADP-ribosyltransferases ARTD8 and ARTD9 as novel oncogenic survival factors in androgen-independent mPCa cells. Constitutive overexpression of DTX3L and ARTD9 is mediated through both IL6/JAK1-STAT1:STAT3- and IFNγ/JAK1-STAT1:STAT1-mediated signaling pathways (Figure 9A). Together with ARTD8 and ARTD9, DTX3L mediates proliferation, chemo-resistance and survival in mPCa cells (Figure 9B). Our study demonstrates that DTX3L and ARTD9 cooperate as repressors of the tumor suppressor IRF1 in mPCa cells (Figure 9C). However, since depletion of IRF1 does only positively affect proliferation but not cell survival, the DTX3L/ARTD9-mediated effects on survival observed in the mPCa cell lines used in this study are only partially dependent on IRF1 in these cells. Thus, the DTX3L/ARTD8 and DTX3L/ARTD9 target genes, which act together with IRF1 in mediating survival and/or proliferation, respectively, remain to be identified in future studies.Our results suggest that both DTX3L and ARTD9 may influence the nuclear activities of both STAT1α and STAT1β by antagonistically regulating the tyrosine phosphorylation of STAT1 on Y701 and therefore being required for the fine-tuning of STAT1-signaling, particularly in tumorigenesis (Figure 9C). Conversely, both DTX3L and ARTD9 cooperate in the transcriptional repression of IRF1. Thus, the exact molecular mechanisms are much more complicated and remain to be elucidated in future studies.

In addition to their regulatory roles in STAT1-mediated chemo-resistance, both DTX3L and ARTD9 could also be directly involved in editing or inhibiting the IFNγ-dependent host immune response against tumor cells through the termination of IFNγ-mediated gene expression and the inhibition of the extrinsic IFNγ-induced anti-proliferative and pro-apoptotic STAT1-IRF1-X-axis. Alternatively, the observed crosstalk between DTX3L/ARTD9 and ARTD8 in absence of IFNγ strongly indicates that DTX3L/ARTD9 and ARTD8 act independently of IFNγ-mediated signaling in cell proliferation and survival.

Our data provide first evidence for a crosstalk between mono-ubiquitin-ligase(s) and mono-ADP-ribosyltransferases that mediates proliferation and survival in mPCa and thus suggest that these processes might be tightly regulated by mono-ADP-ribosylation and (mono)-ubiquitination. However, the potential (mono)-ubiquitinylation activity of DTX3L and the exact molecular mechanisms of ARTD8-mediated mono-ADP-ribosylation underlying the observed crosstalk remain to be addressed in future studies.

Our *in vitro* study suggests that DTX3L together with STAT1 might be required for the metastasis and dissemination of metastatic CRPC cells *in vivo* (Figure 9D). Thus, further studies need to be carried out in order to determine whether simultaneous ectopic co-overexpression of ARTD9 together with wild type or enzymatic mutant forms of DTX3L and/or ARTD8 in xenograft prostate tumors confer docetaxel resistance and/or enhance metastasis *in vivo*.

Taken together, our study suggests that the combined targeted inhibition of STAT1, ARTD8, ARTD9 and/or DTX3L could increase the efficacy of chemotherapy or radiation treatment in prostate and other high-risk tumor types with an increased STAT1- and STAT3-signaling. For instance, the combination of classical therapeutic drugs with highly ARTD8 or DTX3L-specific inhibitors and drugs specifically targeting STAT1 or the macrodomains of ARTD9 might provide a novel therapeutic strategy to increase the sensitivity of PCa cells towards classical therapy, and thus pave the way to develop novel personalized therapeutic strategies for patients suffering from aggressive PCa.

## Material and methods

### Cell culture, transfections, luciferase reporter assays and generation of stable cell lines

The CRPC-like mPCa cell lines PC3 and DU145 [54,61] as well as the JAK1-negative, poorly tumorigenic cell line LNCaP [54,61,67,68] were all purchased from ATCC (American Type Culture Collection). They were cultured in 50% Ham’s-F12 and 50% of RPMI-1640, Glutamax-I, 10 mmol/l HEPES with 10% FCS, and Penicillin and Streptomycin. Transfections of cells with plasmid DNA were performed with Fugene HD, Extreme gene 9 and HP transfection reagents (Roche Applied Science) according to the manufacturers’ protocols. Transfections of siRNA oligos were performed with Lipofectamine RNAimax (Invitrogen) or Extreme gene siRNA reagents (Roche Applied Science) according to manufacturers’ protocols. For complementation of PC3-siARTD8 knockdown cells with non-degradable cDNAs of active ARTD8 wild type or catalytically inactive ARTD8 mutant form, transfections of cells with cDNAs were performed 24 h after transfection of siRNA oligos. Cells were generally treated/pretreated with siRNA oligos for 36-48 h before the assays were performed.

### Plasmids

Human *DTX3L* cDNA was amplified by PCR from a B-cell Lymphoma cDNA library and cloned into the corresponding expression vectors (*EF1a*-promoter driven) using BamHI-NotI respectively. The mouse *ARTD8* cDNA was a generous gift from Dr. M. Boothby (Vanderbilt University School of Medicine, Nashville, TN, USA) and cloned into the corresponding expression vectors (*EF1a*-promoter driven) using BamHI-NotI respectively. The enzymatically inactive ARTD8 mutant form containing two mutations in the evolutionary conserved catalytic triad motif (H-Y-I < - > Q-Y-T; aa 1698H-Q and aa1798I-T) [38,96] was generated by PCR and verified by sequencing. The siRNA oligos were purchased from Qiagen. The corresponding siRNA sequences are listed in Additional file 11: Table S1. Expression vectors for STAT1 and ARTD9 are described in [23]. Expression vectors for human IRF1 were purchased from Addgene. *hIRF1*-prom-luciferase reporter vectors were a nice gift from Dr. R. Pine (Public Health Research Institute, Newark, NJ, USA).

### Reagents

Human recombinant interferons were all purchased from PeproTech or kindly provided by Dr. J. Pavlovic (Institute of Medical Virology, University of Zurich, Switzerland), docetaxel and doxorubicin were purchased from SIGMA. Tosyl-activated Dynabeads were purchased from Invitrogen. ADP-ribose was purchased from SIGMA.

### Interaction assays, immunoblot analyses and immunofluorescence microscopy

Membrane, cytoplasmic, nuclear, and whole cell extracts were prepared as described in [23,97,98]. For immunoprecipitation membrane and cytoplasmic extract fractions were re-mixed. Co-immunoprecipitation assays were performed as described previously [23,97,98] using the following DTX3L and ARTD9 specific antibodies: rabbit anti-DTX3L antibody Cat.No.: D9644-01B), rabbit anti-DTX3L antibody (Bethyl Laboratories, Inc., Cat.No.: A300-833A,) and rabbit anti-ARTD9 antibody (Chemicon/EMD Millipore, Cat.No.: AB10619, Lot No.: LV1409682). All antibodies used for immunoprecipitation analysis were covalently coupled to tosyl-activated Dynabeads (Invitrogen) according to the manufacturers’ protocols. Immunoblot analysis and immunofluorescence microscopy were performed as described in [23,97,98] using the following primary antibodies: Rabbit anti-DTX3L (US Biological, Cat.No.: D9644-01B), rabbit anti-DTX3L, (Bethyl Laboratories, Inc., Cat.No.: A300-833A), rabbit anti-ARTD9 (EMD Millipore, Cat.No.: AB10618), rabbit anti-ARTD9 antibody (EMD Millipore, Cat.No.: AB10619), mouse anti-ARTD2 (EMD Millipore, Cat.No.: MABE18), rabbit anti-ARTD3 (Aviva Systems Biology Corp., Cat.No.: OAAB03449), rabbit anti-ARTD10 (Aviva Systems Biology Corp., Cat.No.: ARP42810_P050), rabbit anti-ARTD12 (Aviva Systems Biology Corp., Cat.No.: OAAB03451), rabbit anti-ARTD11 (Abgent, Cat.No.: AP6297a), rabbit anti-ARTD13 (GeneTex, Cat.No.: N3C2), anti-STAT1α/β (RabMab, Epitomics, Cat.No.: 2728–1), anti-pSTAT1α/β(Y701) (RabMab, Epitomics, Cat.No.: 2825–1), anti-pSTAT1α(S727) (RabMab, Epitomics, Cat.No.: 3324–1), anti-STAT2 (RabMab, Epitomics, Cat.No.: 1513–1), anti-STAT3α (RabMab, Epitomics, Cat.No.: 3566–1), anti-pSTAT3α(S727) (RabMab, Epitomics, Cat.No.: 2236–1), anti-STAT5 (RabMab, Epitomics, Cat.No.: 1289–1), anti-pSTAT5(S726) (RabMab, Epitomics, Cat.No.: 5734–1), anti-STAT6 (RabMab, Epitomics, Cat.No.: 1505–1), anti-PTPN1 (RabMab, Epitomics, Cat.No.: 3774–1), anti-PTPN2 (RabMab, Epitomics, Cat.No.: 5790–1), anti-pJAK1 (RabMab, Epitomics, Cat.No.: 6518–1), anti-JAK1 (RabMab, Epitomics, Cat.No.: 2856–1), anti-IFNGR1 (RabMab, Epitomics, Cat.No.: 5697–1), anti-IFNGR2 (RabMab, Epitomics, Cat.No.: 7932–1), anti-IRF1 (RabMab, Cell Signaling Technology, Cat.No.: 8478), anti-STAT3α/β (RabMab, Cell Signaling Technology, Cat.No.: 12640), rabbit anti-pSTAT2(Y690) (St. Cruz Biotechnology, Inc., Cat.No.: sc-21689-R), rabbit anti-pSTAT6(Y641) (St. Cruz Biotechnology, Inc., Cat.No.: sc-101808) and mouse anti-tubulin (SIGMA, Cat.No.: T5618). The rabbit anti-ARTD8 antibody was a generous gift from Dr. Avraham Raz (Karmanos Cancer Institute, School of Medicine, Wayne State University, Detroit, Michigan 48201, USA [99]). Immunofluorescence analysis was performed with an automated inverted research microscope system (Leica DMI6000B, Leica Microsystem). Composite images were generated by Adobe Photoshop software. Quantification of immunoblots was performed using the GelEval software (FrogDance Software Inc.) and mean value ± SE was calculated and plotted into graphs using the GraphPad Prism 5 software (GraphPad Software, Inc.).

### Survival and proliferation assays

Cell viability and proliferation was assessed by trypan blue exclusion assays as described in [23]. For the cell viability and proliferation assays cells were seeded at 0.2 × 10^6^ cells/well (PC3 and DU145) and 0.1 × 10^5^ cells/well (LNCaP) in 6 well dishes 8-12 h prior to initiation of treatment and then incubated for 24 h in the presence of PBS, DMSO (mock-treated), IFNγ (200 U/ml) or docataxel (0.5-1 nM), ARTD/PARP inhibitors Olaparib (1 μM), Veliparib (1 μM), DPQ or TIQ-A (7.5 μM). Relative cell viability/proliferation and cell numbers are presented as means from three (PC3 and DU145) or two (LNCaP) independent experiments performed in triplicates ± SE. All data were analyzed with Excel (Microsoft Inc.) and GraphPad Prism 5 software. Analyzed data were plotted into graphs using the GraphPad Prism 5 software (GraphPad Software, Inc.).

### Gene expression analysis

Total RNA was isolated using Trizol (Invitrogen) or Tri-Reagent (MRC, Inc) according to manufacturer’s protocols. RNA was subsequently reverse-transcribed using the ‘High-capacity cDNA reverse transcription kit (Applied Biosystems) according to manufacturer’s protocols. Real-time (RT) qPCR was performed using the Rotor-Gene 3000 (Corbett Life Science, now Qiagen) and SYBR Green kit (Bioline) according to manufacturer’s protocols using the RT-qPCR primers listed in Additional file 12: Table S2. Mean value ± SE was calculated and blotted into graphs with GraphPad Prism 5 software (GraphPad Software). Q-RT-PCR Primer sequences are shown in Additional file 12: Table S2.

### Luciferase reporter assays

Luciferase reporter assays were performed as previously described [97] and according to manufacturer’s protocol (Promega) using the dual luciferase assay kit (Promega) and a TECAN infinite M200 luminometer (Tecan Systems). Briefly, PC3 cells were seeded in 6-well plates at 0.4 × 10^6^ cells/well and co-transfected with an *IRF1*-promoter-driven luciferase reporter vector (500 ng DNA/ml) along with expression vectors for DTX3L, ARTD9 and/or STAT1α/β (800 ng DNA/ml) and with the control reporter plasmid, pRL-hTK (100 ng/ml) (*hTK-*prom-renilla–luciferase control), and subsequently treated with or without IFNγ (200 U/ml) for 4 h. *IRF1*-promoter-luciferase activities were normalized to the luciferase activities of the internal *hTK-*prom-renilla-luciferase control and presented as mean from five independent experiments performed in triplicates. Statistical analysis was performed using the Student's t test. *P < 0.05, **P < 0.001 and ***P < 0.0001. For siRNA knockdown experiments, PC3 cells were co-transfected in serious: first with mock-siRNA, STAT1-siRNA, DTX3L-siRNA or ARTD9-siRNA and subsequently (24 h later) with an *IRF1*-promoter-driven luciferase reporter vector (500 ng DNA/ml) along with expression vectors for DTX3L, ARTD9 and/or STAT1α/β (800 ng DNA/ml) and with the control reporter plasmid, pRL-hTK (100 ng DNA/ml).

### Scratch wound healing migration assay

DU145 or PC3 cells were seeded into 6-well plates (0.2 × 10^6^ cells/well) and transfected with siRNA as indicated. After 24 h the cells were trypsinized and 400’000 cells were pooled into one well. After 24–36 h when cells reached confluency, identical scratches were made in parallel wells using a 1000 μl plastic pipette tip. Non-adherent cells were removed by two washes. The closure of the scratch was analyzed under the microscope and images were captured at 0, 12, 24, and 36 h after incubation. Photographs were made with a Leica DMI6000B automated inverted research microscope system (Leica Microsystems) at indicated time points. The size of the uncovered areas was measured with Adobe Photoshop software and converted into percentages. For analysis of the migration potential mean values of three independent experiments were analyzed. Mean value ± SE was calculated and plotted into graphs with GraphPad Prism 5 software (GraphPad Software, Inc.).

### Statistical analysis

Continuous variables were summarized as mean and SE. Statistical evaluations (comparisons between control and treated groups) were established by Student's T-test for unpaired data (for two comparisons). P values < 0.05 were considered statistically significant. All statistical evaluations were performed with GraphPad Prism 5 software (GraphPad Software, Inc.).

### Availability of supporting data

“The data set(s) supporting the results of this article is (are) included within the article (and its additional file(s))”.

## Competing interests

The authors declare no competing financial and non-financial interests.

## Authors’ contributions

Contribution: SBB, SCF, RC, HCW and POH designed the experiments and analyzed results. SBB, RC, HCW, SCF, RS and POH performed the research; SBB, SCF and POH wrote the paper. POH designed and supervised the research study. All the authors read and corrected the manuscript. All authors read and approved the final manuscript.

## Supplementary Material

Additional file 1: Figure S1 Quantification of ARTD9, DTX3L, IRF1, STAT1 and pSTAT1 protein levels. **(A)** Quantification of ARTD9 and DTX3L protein levels in PC3, DU145 and LNCaP cells, represented in Figure 1B. ARTD9 and DTX3L protein levels were normalized to tubulin. **(B)** Quantification of pSTAT1-Y701 and pSTAT1-S727 protein levels in PC3, DU145 and LNCaP cells, represented in Figure 1B. pSTAT1-Y701 and pSTAT1-S727 protein levels were normalized to tubulin and STAT1. All values represent the means of three independent experiments, and the error bars represent the SE. Statistical analysis was performed using the Student's t test. *P < 0.05, **P < 0.001 and ***P < 0.0001. **(C)** Immunoblot analyses of STAT signaling in PC3, DU145 and LNCaP cells treated with or without IFNγ (200 U/ml) or IFNαβ (50 U/ml each). Whole cell extracts were separated by SDS PAGE and subsequently probed with antibodies for STAT1αβ, pSTAT1(Y701), STAT2, pSTAT2(Y690), STAT3α, STAT3αβ, pSTAT3α(S727), STAT5αβ, pSTAT5(S726), STAT6 and pSTAT6(Y641) and tubulin. **(D)** Immunoblot analyses of ARTD8, ARTD9 and DTX3L levels in PC3-siMock and PC3-siJAK1 cells. Whole cell extracts were separated by SDS PAGE, blotted and subsequently probed with antibodies for JAK1, ARTD8, ARTD9, DTX3L and tubulin. (**D** right panel) Analysis of JAK1- siRNA knockdown efficiency in PC3 cells; JAK1 protein levels were normalized to tubulin. **(E)** Immunoblot analyses of ARTD9 and DTX3L protein levels in PC3-siMock and PC3-siSTAT3 cells. Whole cell extracts were separated by SDS PAGE, blotted and subsequently probed with antibodies for ARTD9, DTX3L and tubulin. All immunoblots are representative of at least three independent experiments. (**E** right panel) Analysis of STAT3-siRNA knockdown efficiency in PC3 cells; Total RNA was isolated from PC3-siMock, and PC3-siSTAT3 cells and STAT3 mRNA levels were measured by RT-qPCR, normalized against GAPDH and presented as mean from three independent experiments performed in triplicate ± SE. Click here for file

Additional file 2: Figure S2 Sub-cellular localization of endogenous STAT1 in DU145 and LNCaP cells and quantification of IRF1 protein levels in PC3, DU145 and LNCaP cells. **(A)** Immunofluorescence microscopy analyses and sub-cellular localization of endogenous STAT1, pSTAT1-(pY701) and pSTAT1-(pS727) in DU145 cells, in presence or absence of 1000 U/ml IFNγ. Original magnification × 400. Images are representative of at least three independent experiments. **(B)** Immunofluorescence microscopy analyses and sub-cellular localization of endogenous STAT1, pSTAT1-(pY701) and pSTAT1-(pS727) in LNCaP cells. Original magnification × 400. Images are representative of at least three independent experiments. **(C)** Quantification of IRF1 protein levels in PC3, DU145 and LNCaP cells, as represented in Figure 1C. IRF1 levels were normalized to tubulin. Values represent the means of three independent experiments, and the error bars represent the SE. Statistical analysis was performed using the Student's t test. *P < 0.05, **P < 0.001 and ***P < 0.0001, according to the t-test analysis. Click here for file

Additional file 3: Figure S3 Sub-cellular localization of endogenous DTX3L and ARTD9 in PC3-siARTD9 or -siDTX3L knockdown cells, respectively. **(A)** Immunofluorescence microscopy analyses and sub-cellular localization of endogenous DTX3L and ARTD9 in PC3-siMock **(A)**, PC3-siDTX3L **(B)** and PC3-siARTD9 **(C)** knockdown cells in absence or presence of IFNγ (200 U/ml). Original magnification × 400. Images are representative of at least three independent experiments. Click here for file

Additional file 4: Figure S4 Co-staining of endogenous DTX3L and ARTD9 in PC3-siARTD9 or -siDTX3L knockdown cells, respectively. **(A)** Co-staining and immunofluorescence microscopy analyses of endogenous DTX3L and ARTD9 in PC3-siMock **(A)**, PC3-siDTX3L **(B)** and PC3-siARTD9 **(C)** knockdown cells in absence or presence of IFNγ (200 U/ml). Cells were co-stained using a mouse monoclonal anti-DTX3L antibody (red) together with a rabbit polyclonal anti-ARTD9 antibody (green). Original magnification × 400. Click here for file

Additional file 5: Figure S5 Quantifications of ARTD8-, ARTD9- and DTX3L-siRNA knockdown efficiencies and analysis of ARTD8, ARTD9 and DTX3L containing complexes. **(A and B)** Analysis of ARTD8, ARTD9 and DTX3L-siRNA knockdown efficiency in PC3 cells. **(A)** Gene expression analysis of ARTD8, ARTD9 and DTX3L in PC3-siMock, PC3-siARTD8, PC3-siARTD9 and PC3-siDTX3L cells, respectively. ARTD8, ARTD9 and DTX3L mRNA levels were measured by RT-qPCR, normalized against GAPDH and presented as mean from three independent experiments performed in triplicate ± SE. **(B)** Quantification of ARTD8, ARTD9 and DTX3L protein levels in in PC3-siMock, PC3-siARTD8, PC3-siARTD9 and PC3-siDTX3L cells, respectively. ARTD8, ARTD9 and DTX3L levels were normalized to tubulin. Values represent the means of three independent experiments, and the error bars represent the SE. **(C)** Co-immunoprecipitation control analyses to confirm the specificity of the anti- DTX3L antibody. **(D)** Interactions of endogenous ARDT8 with ARTDs but not with DTX3L are mediated by (mono)-ADP-ribosylation. Endogenous ARTD8-ARTDx and ARTD8-DTX3L complexes from PC3 cell extracts were co-immunoprecipitated in presence or absence of 5 mM mono-ADP-ribose using epitope affinity purified anti-ARTD8 antibodies. Complexes were then separated on SDS PAGE, blotted and subsequently probed with antibodies against endogenous ARTD1, ARTD8, ARTD9, ARTD10 and DTX3L. ARTD1 was used as a positive control for ARTD8 and ARTD9 [80] and ARTD10 was used as a positive control for ARTD8 [44]. **(E)** Interactions of endogenous ARDT9 with ARTDs are mediated by (mono)-ADP-ribosylation. PC3 cells were stimulated for 1 h with IFNγ (200 U/ml) and endogenous ARTD9-ARTDx complexes subsequently co-immunoprecipitated in presence or absence of 5 mM mono-ADP-ribose using epitope affinity purified anti-ARTD9 antibodies. Complexes were then separated on SDS PAGE, blotted and subsequently probed with antibodies against endogenous ARTD9, ARTD10, ARTD12 (also known as PARP12) and ARTD13 (also known as PARP13, ZAPS/L). **(F)** Co-immunoprecipitation control analyses to confirm the specificity of the anti-ARTD9 antibody. Click here for file

Additional file 6: Figure S6 Cell viability and proliferation analyses of siDTX3L, siARTD8 and siARTD9 single knockdown cells. **(A and B)** Cell viability analyses of PC3-siMock, PC3-siDTX3L cells silenced with si*1 or si*2 RNA oligos **(A)** and PC3-siARTD9 cells silenced with si*1 or si*9 RNA oligos **(B)** were assessed by the trypan blue exclusion assay. Cells were treated as indicated with IFNγ and/or docetaxel (DT) and counted after 48 h. Values represent the means of three independent experiments, and the error bars represent the SE. **(C and D)** Immunoblot analyses of ARTD9- and DTX3L-siRNA knockdown efficiencies in DU145 cells. Whole cell extracts were separated by SDS PAGE, blotted and subsequently probed with antibodies for ARTD9 **(C)**, DTX3L **(D)** and tubulin. **(E)** Cell proliferation analyses of DU145-siMock, DU145-siDTX3L and DU145-siARTD9 single knockdown cells in presence or absence of IFNγ (200 U/ml) was assessed by the trypan blue exclusion assay. **(F)** Cell viability analyses of DU145-siMock, DU145-siDTX3L and DU145-siARTD9 knockdown cells were assessed by the trypan blue exclusion assay. Cells were treated as indicated with IFNγ and/or docetaxel (DT) and counted after 48 h, NT: not treated, CT: control treatment (solvent). **(G)** Survival of PC3-siMock or PC3- siARTD8 knockdown cells, complemented with non-degradable mouse cDNAs of active ARTD8 wild type or catalytically inactive ARTD8 mutant form, respectively, were assessed by the trypan blue exclusion assay. Cells were treated as indicated with docetaxel (DT) and/or with the ARTD inhibitor TIQ, CT: control treatment (solvent), EV: empty vector control. All Values shown in **E** to **G** represent the means of three independent experiments performed in triplicates, and the error bars represent the SE. Statistical analysis was performed using the Student's t test. *P < 0.05, **P < 0.001 and ***P < 0.0001. Click here for file

Additional file 7: Figure S7 Quantifications of STAT1-siRNA knockdown efficiencies and *IRF1* promoter analysis **(A and B)**. Analysis of STAT1-siRNA efficiency in PC3 cells. **(A)** Gene expression analysis of STAT1 in PC3-siMock and PC3-siSTAT1 knockdown cells; Total RNA was isolated from PC3-siMock and PC3-siSTAT1 knockdown cells and STAT1 mRNA levels were measured by RT-qPCR and normalized against GAPDH. **(B)** Quantification of STAT1 protein levels in PC3-siMock and PC3-siSTAT1 knockdown cells; STAT1 levels were normalized to tubulin. **(C)** Gene expression analysis of IRF1 in PC3-siMock, PC3-siARTD9 and PC3-siDTX3L knockdown cells. Total RNA was isolated from PC3-siMock, PC3-siARTD9 and PC3-siDTX3L knockdown cells and IRF1 mRNA levels were measured by RT-qPCR and normalized against GAPDH. All Values shown in **A** to **C** represent the means of three independent experiments, and the error bars represent the SE. **(D)** DTX3L- iRNA and ARTD9-siRNA mediated activation of the *IRF1*-promoter driven luciferase in PC3 cells. PC3 cells were co-transfected in series with mock-siRNA, STAT1-siRNA, DTX3L-siRNA or ARTD9-siRNA and plasmids for an *IRF1*-promoter-driven luciferase reporter vector as described in Material and Methods and subsequently treated with or without IFNγ (200 U/ml) for 4 h. **(E-G)** DTX3L and ARTD9 together with STAT1β inhibit the *IRF1*-promoter driven luciferase in PC3 cells. PC3 cells were co-transfected with an *IRF1*-promoter-driven luciferase reporter vector along with expression vectors for DTX3L, ARTD9 and/or STAT1α/β and subsequently treated with or without IFNγ (200 U/ml) for 4 h. *IRF1*-promoter-luciferase activities shown in D to G are presented as mean from five independent experiments performed in triplicates. The error bar represents the SE. Statistical analysis was performed using the Student's t test. *P < 0.05, **P < 0.001 and ***P < 0.0001. Click here for file

Additional file 8: Figure S8 Quantifications of IRF1 protein levels, quantifications of IRF1-siRNA knockdown efficiencies and cell viability analysis of siIRF1 knockdown cells. **(A)** Immunoblot analyses of IRF1 protein levels in PC3-CMVprom-empty-control and PC3-CMVprom-IRF1 cells. Whole cell extracts of PC3-CMVprom-empty-control and PC3-CMVprom7 IRF1 cells were separated by SDS PAGE, blotted and probed with antibodies for IRF1 and tubulin. (**A** right panel) Quantification of IRF1 protein levels in PC3-CMVprom-empty-control and PC3-CMVprom-IRF1 cells; IRF1 levels were normalized to tubulin. **(B and C)** Analysis of IRF1- siRNA efficiency in PC3 cells. **(B)** Immunoblot analyses of IRF1 protein levels in PC3-siMock and PC3-siIRF1 cells. Whole cell extracts of PC3-siMock and PC3-siIRF1 cells were separated by SDS PAGE, blotted and probed with antibodies for IRF1 and tubulin. (**B** right panel) Quantification of IRF1 protein levels in PC3-simock and PC3-siIRF1 cells; IRF1 levels were normalized to tubulin. **(C)** Gene expression analysis of IRF1 in PC3-siMock and PC3-siIRF1 knockdown cells. IRF1 mRNA levels were measured by RT-qPCR and normalized against GAPDH. **(D)** Gene expression analysis of IRF1 in LNCaP-siMock and LNCaP-siIRF1 knockdown cells. IRF1 mRNA levels were measured by RT-qPCR and normalized against GAPDH. **(E)** Cell viability analyses of PC3-siMock, PC3-siIRF1, PC3-siARTD9 and PC3-siARTD9/siIRF1 cells were assessed by the trypan blue exclusion assay. Cells were treated as indicated with 50 ng/ml IFNγ and 0.25 μM doxorubicin **(D)** and counted after 48 h and 72 h, respectively, NT: not treated, CT: control treatment (solvent). All values shown in A to E represent the means of three independent experiments, and the error bars represent the SE. Statistical analysis was performed using the Student's t test. *P < 0.05, **P < 0.001 and ***P < 0.0001. **(F)** Co-immunoprecipitation analyses of endogenous nuclear DTX3L/ARTD9/STAT1 complexes, respectively in PC3 cells. Endogenous STAT1, DTX3L or RTD9 complexes were co-immunoprecipitated from nuclear extracts using anti-ARTD9 antibodies and separated on SDS PAGE, blotted and probed with antibodies for STAT1, DTX3L and ARTD9. Click here for file

Additional file 9: Figure S9 Photographs and quantification of cell migration in siARTD8, siARTD9 and siDTX3L single knockdown prostate cancer cells. **(A-D)** Photographs of cell migration in PC3 prostate cancer cells. PC3-siMock **(A)**, PC3-siDTX3L **(B)**, PC3-siARTD9 **(C)** and PC3-siARTD8 **(D)** single knockdown cells were seeded into 6-well plates and treated as described in Material and Methods. At 0, 12, 24, and 36 h photographs were made. **(E and F)** Quantification of cell migration in DU145 prostate cancer cells. DU145-siDTX3L **(E)** and DU145-siARTD9 **(F)** single knockdown cells were seeded into 6-well plates and treated as described in Material and Methods. At 0, 12, 24, and 36 h photographs were made and quantified as described in Material and Methods. Values represent the mean of three independent experiments and the error bars represent the SE. Statistical analysis was performed using the Student's t test. *P < 0.05, **P < 0.001 and ***P < 0.0001. Click here for file

Additional file 10: Figure S10 Photographs and quantification of cell migration in single, double and triple knockdown prostate cancer cells. **(A-C)** Photographs of cell migration in PC3 prostate cancer cells. PC3-siSTAT1 **(A)** PC3-siDTX3L/siSTAT1 **(B)** and PC3-siSTAT3 **(C)** single knockdown cells were seeded into 6-well plates and treated as described in Material and Methods. At 0, 12, 24, and 36 h photographs were made. **(D-F)** Quantification of cell migration in PC3 and DU145 prostate cancer cells. PC3-siIRF1 **(D)**, DU145-siSTAT1 **(E)** and DU145-siSTAT3 **(F)** single knockdown cells, DU145-siSTAT1/siSTAT3 **(G)** double knockdown cells and DU145-siDTX3L/siSTAT1/siSTAT3 (H) triple knockdown cells were seeded into 6-well plates and treated as described in Material and Methods. At 0, 12, 24, and 36 h photographs were made and quantified as described in Material and Methods. Values represent the mean of three independent experiments and the error bars represent the SE. Statistical analysis was performed using the Student's t test. *P < 0.05, **P < 0.001 and ***P < 0.0001. Click here for file

Additional file 11: Table S1 siRNA sequences. Click here for file

Additional file 12: Table S2 qPCR Primer. Click here for file
