# Supplementary figures and images for: DTX3L and ARTD9 inhibit IRF1 expression and mediate in cooperation with ARTD8 survival and proliferation of metastatic prostate cancer cells
Source: Mol Cancer. 2014 May 27;13:125. doi: 10.1186/1476-4598-13-125 (PMC4070648; doi:10.1186/1476-4598-13-125)

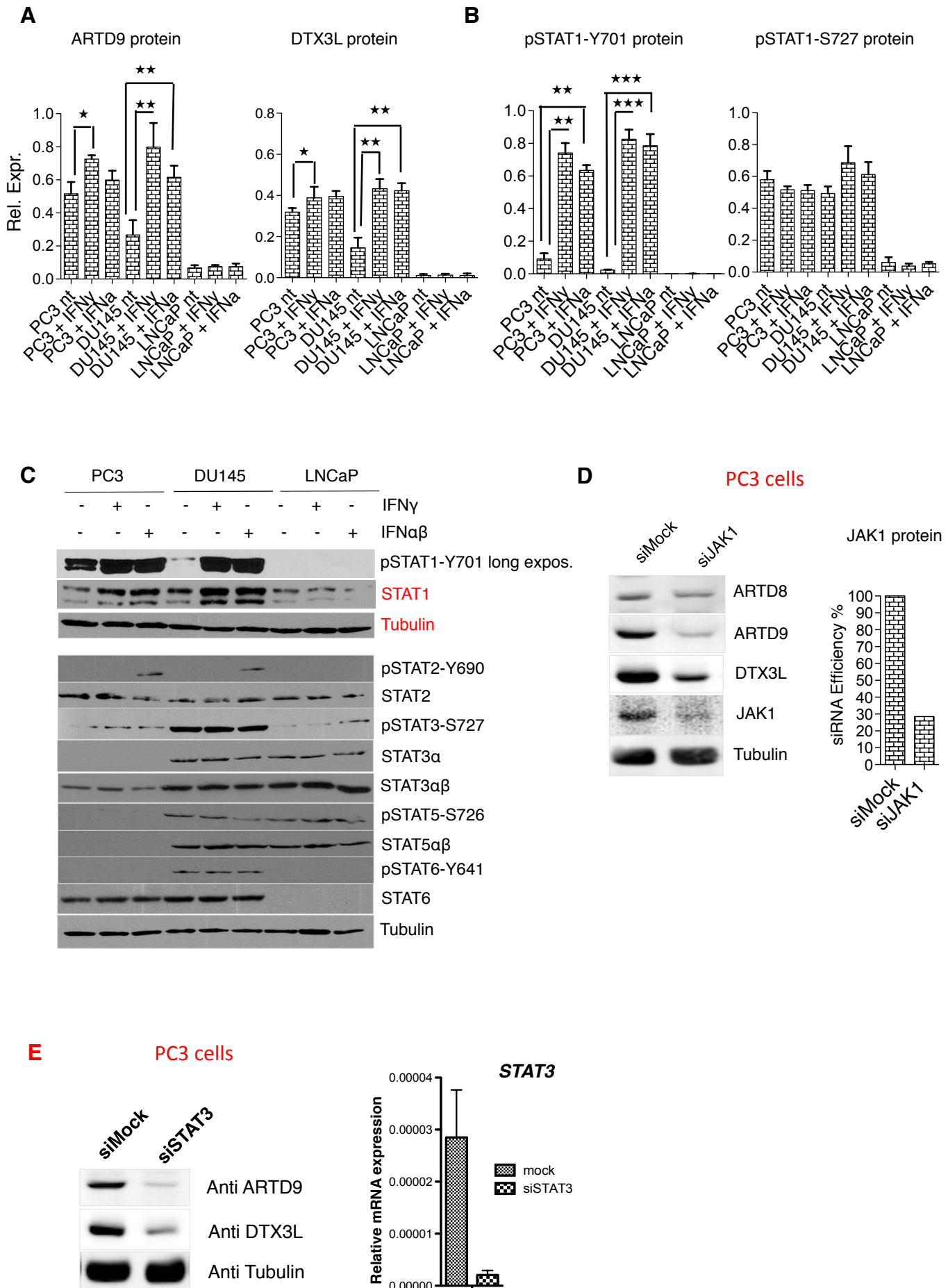

Supplement: Additional file 1: Figure S1 — Quantification of ARTD9, DTX3L, IRF1, STAT1 and pSTAT1 protein levels. (A) Quantification of ARTD9 and DTX3L protein levels in PC3, DU145 and LNCaP cells, represented in Figure 1B. ARTD9 and DTX3L protein levels were normalized to tubulin. (B) Quantification of pSTAT1-Y701 and pSTAT1-S727 protein levels in PC3, DU145 and LNCaP cells, represented in Figure 1B. pSTAT1-Y701 and pSTAT1-S727 protein levels were normalized to tubulin and STAT1. All values represent the means of three independent experiments, and the error bars represent the SE. Statistical analysis was performed using the Student's t test. *P < 0.05, **P < 0.001 and ***P < 0.0001. (C) Immunoblot analyses of STAT signaling in PC3, DU145 and LNCaP cells treated with or without IFNγ (200 U/ml) or IFNαβ (50 U/ml each). Whole cell extracts were separated by SDS PAGE and subsequently probed with antibodies for STAT1αβ, pSTAT1(Y701), STAT2, pSTAT2(Y690), STAT3α, STAT3αβ, pSTAT3α(S727), STAT5αβ, pSTAT5(S726), STAT6 and pSTAT6(Y641) and tubulin. (D) Immunoblot analyses of ARTD8, ARTD9 and DTX3L levels in PC3-siMock and PC3-siJAK1 cells. Whole cell extracts were separated by SDS PAGE, blotted and subsequently probed with antibodies for JAK1, ARTD8, ARTD9, DTX3L and tubulin. (D right panel) Analysis of JAK1- siRNA knockdown efficiency in PC3 cells; JAK1 protein levels were normalized to tubulin. (E) Immunoblot analyses of ARTD9 and DTX3L protein levels in PC3-siMock and PC3-siSTAT3 cells. Whole cell extracts were separated by SDS PAGE, blotted and subsequently probed with antibodies for ARTD9, DTX3L and tubulin. All immunoblots are representative of at least three independent experiments. (E right panel) Analysis of STAT3-siRNA knockdown efficiency in PC3 cells; Total RNA was isolated from PC3-siMock, and PC3-siSTAT3 cells and STAT3 mRNA levels were measured by RT-qPCR, normalized against GAPDH and presented as mean from three independent experiments performed in triplicate ± SE. [file 1476-4598-13-125-S1.pdf]

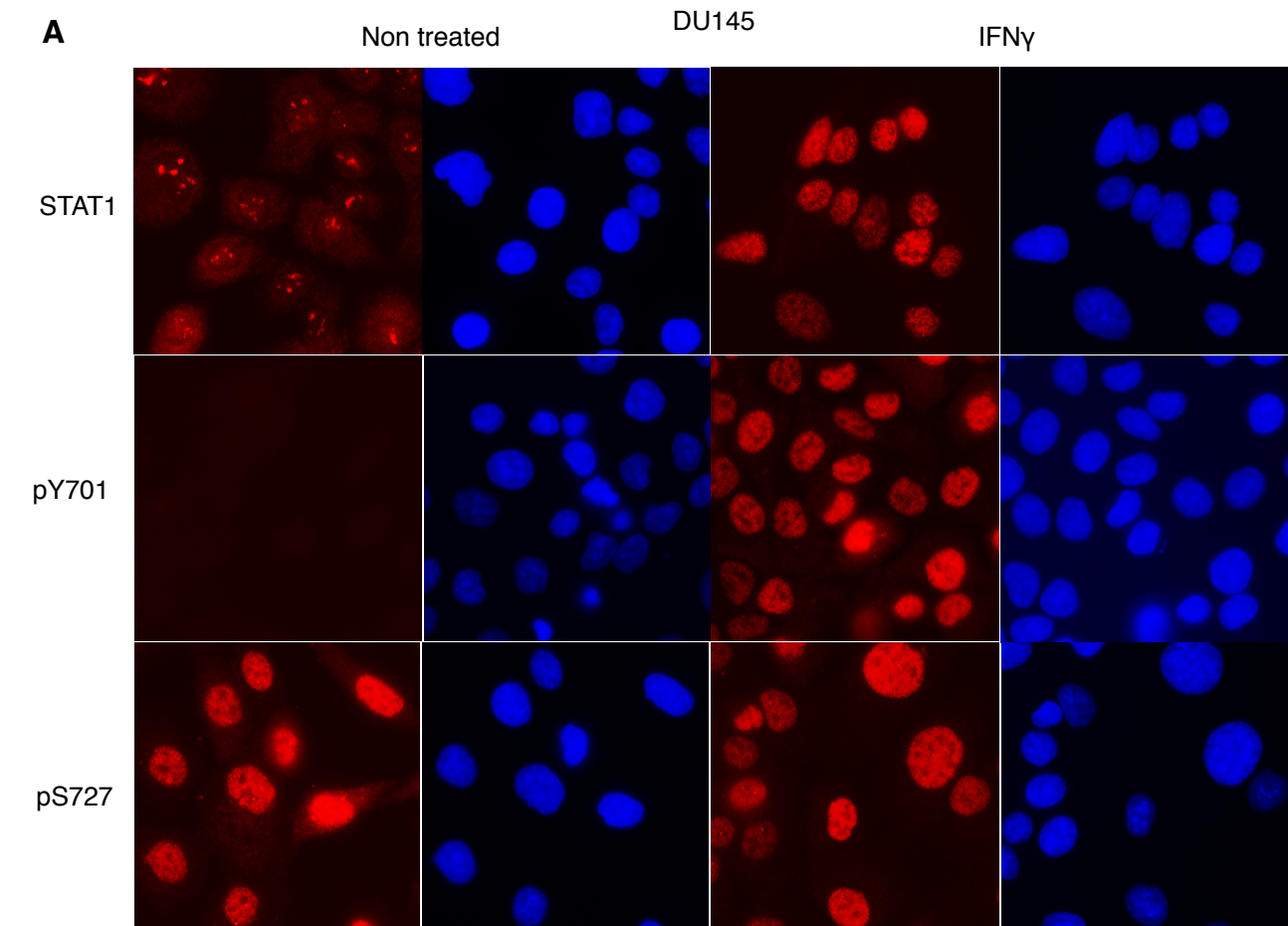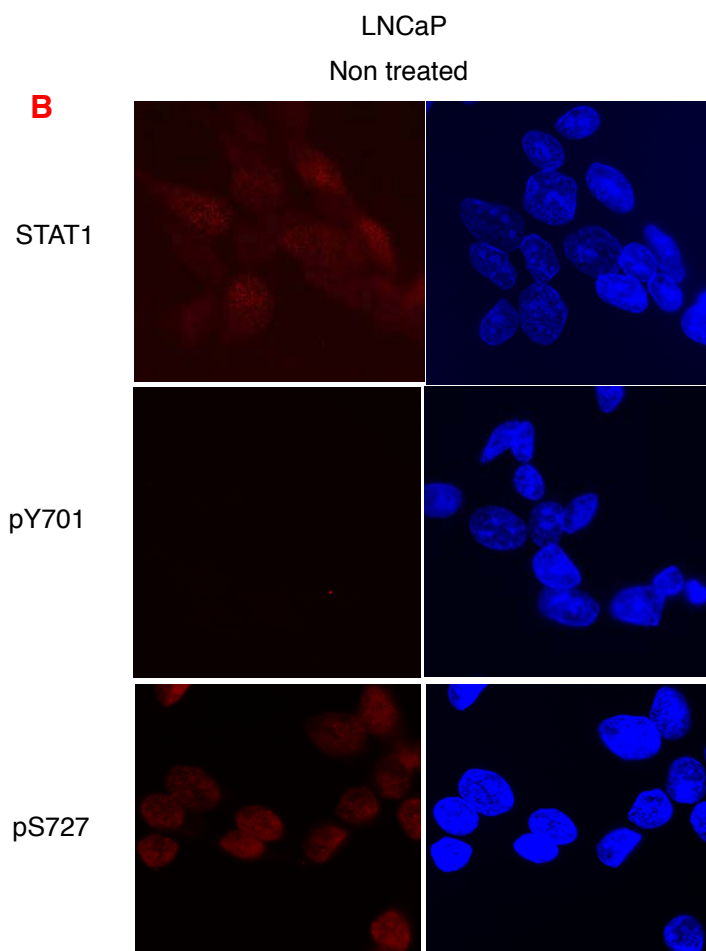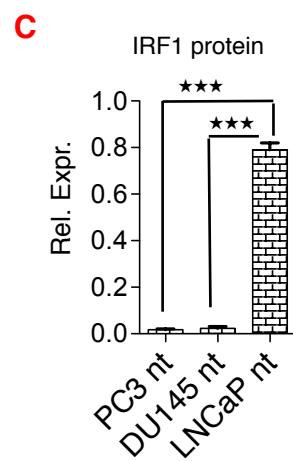

Supplement: Additional file 2: Figure S2 — Sub-cellular localization of endogenous STAT1 in DU145 and LNCaP cells and quantification of IRF1 protein levels in PC3, DU145 and LNCaP cells. (A) Immunofluorescence microscopy analyses and sub-cellular localization of endogenous STAT1, pSTAT1-(pY701) and pSTAT1-(pS727) in DU145 cells, in presence or absence of 1000 U/ml IFNγ. Original magnification × 400. Images are representative of at least three independent experiments. (B) Immunofluorescence microscopy analyses and sub-cellular localization of endogenous STAT1, pSTAT1-(pY701) and pSTAT1-(pS727) in LNCaP cells. Original magnification × 400. Images are representative of at least three independent experiments. (C) Quantification of IRF1 protein levels in PC3, DU145 and LNCaP cells, as represented in Figure 1C. IRF1 levels were normalized to tubulin. Values represent the means of three independent experiments, and the error bars represent the SE. Statistical analysis was performed using the Student's t test. *P < 0.05, **P < 0.001 and ***P < 0.0001, according to the t-test analysis. [file 1476-4598-13-125-S2.pdf]

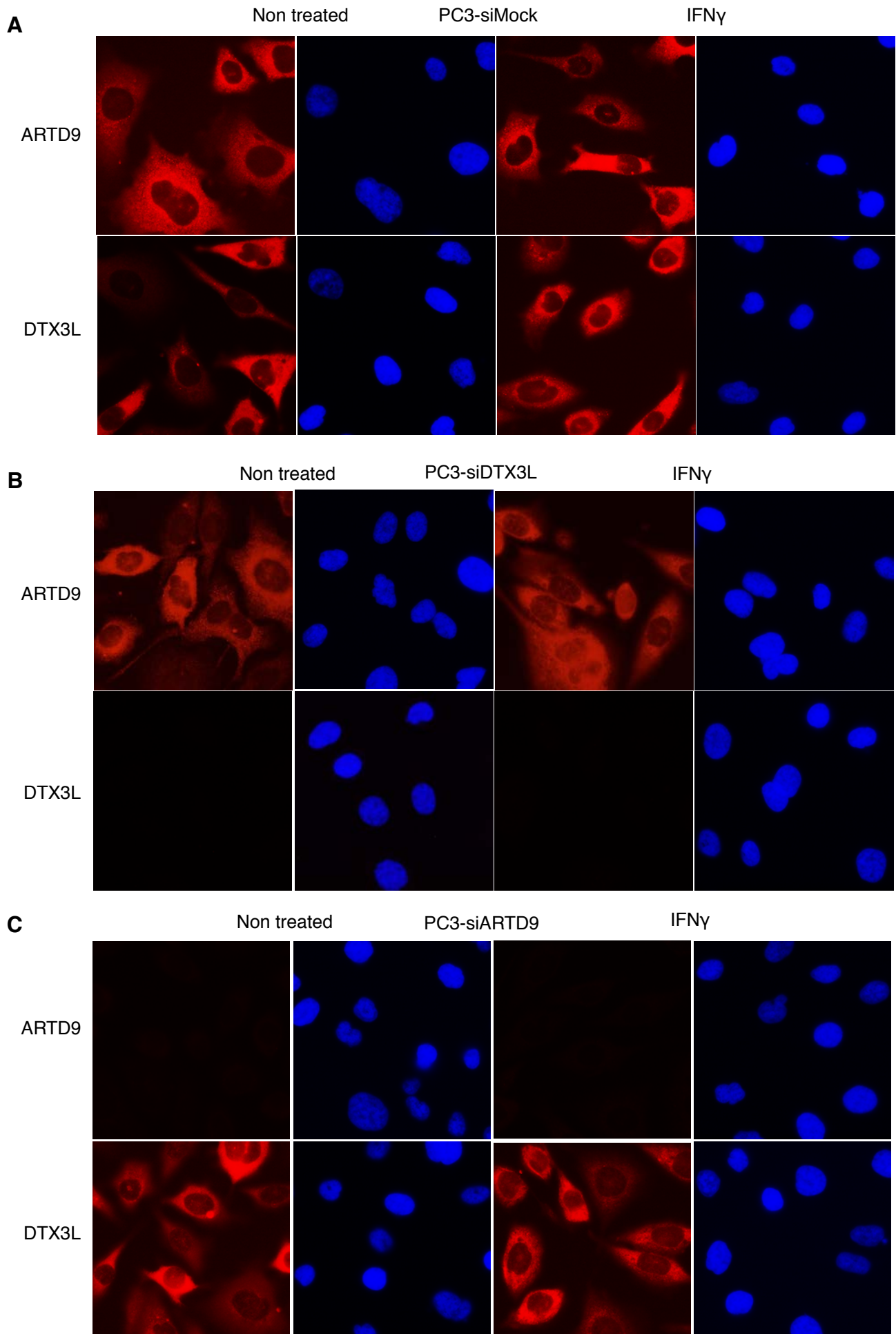

Supplement: Additional file 3: Figure S3 — Sub-cellular localization of endogenous DTX3L and ARTD9 in PC3-siARTD9 or -siDTX3L knockdown cells, respectively. (A) Immunofluorescence microscopy analyses and sub-cellular localization of endogenous DTX3L and ARTD9 in PC3-siMock (A), PC3-siDTX3L (B) and PC3-siARTD9 (C) knockdown cells in absence or presence of IFNγ (200 U/ml). Original magnification × 400. Images are representative of at least three independent experiments. [file 1476-4598-13-125-S3.pdf]

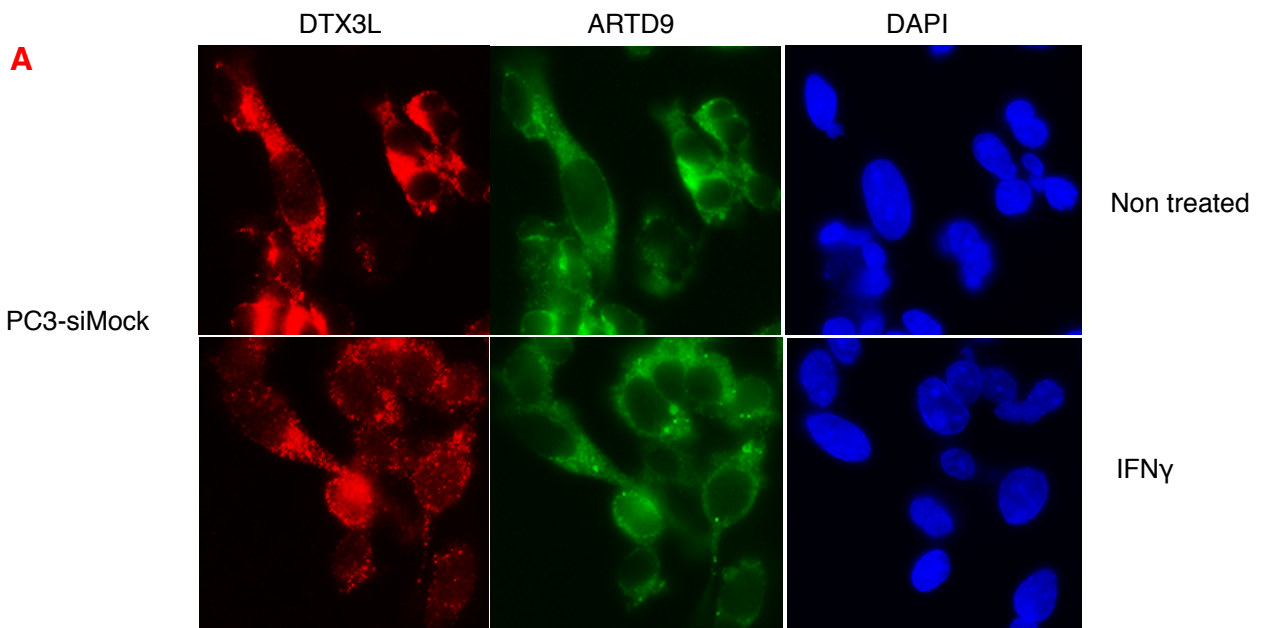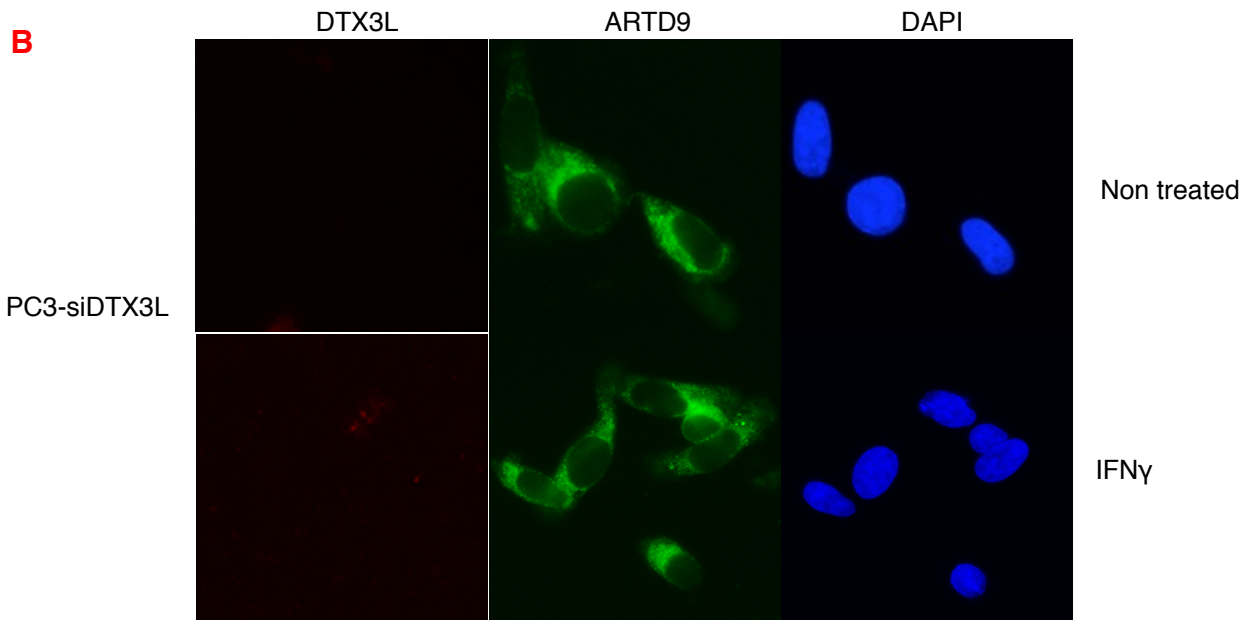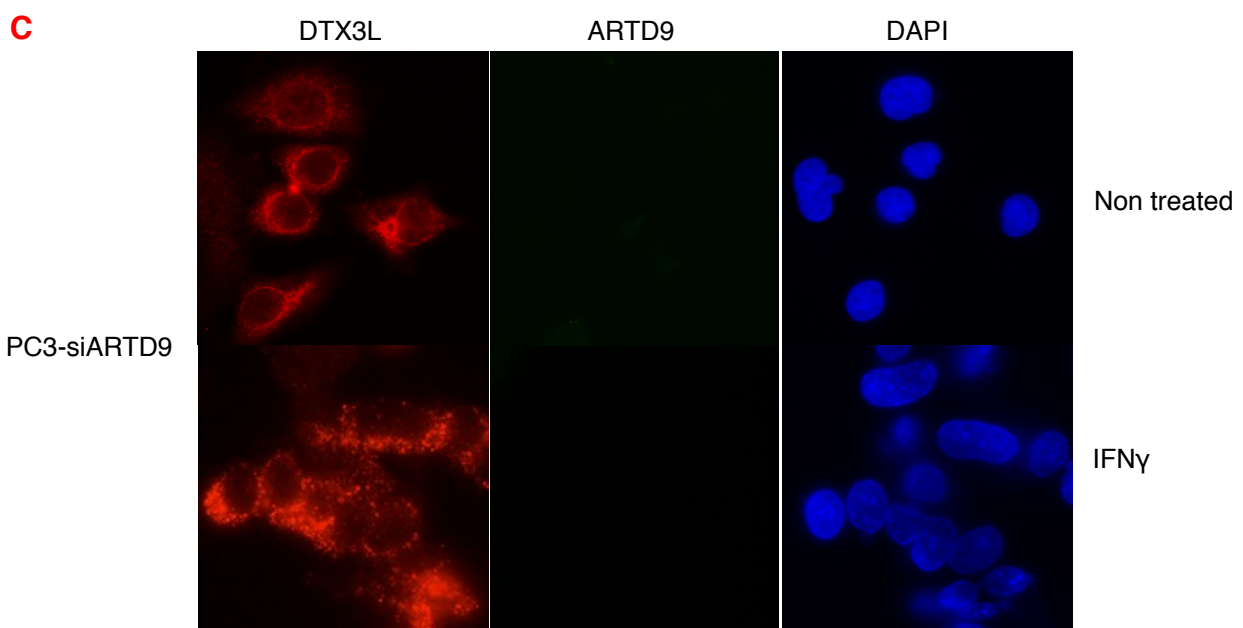

Supplement: Additional file 4: Figure S4 — Co-staining of endogenous DTX3L and ARTD9 in PC3-siARTD9 or -siDTX3L knockdown cells, respectively. (A) Co-staining and immunofluorescence microscopy analyses of endogenous DTX3L and ARTD9 in PC3-siMock (A), PC3-siDTX3L (B) and PC3-siARTD9 (C) knockdown cells in absence or presence of IFNγ (200 U/ml). Cells were co-stained using a mouse monoclonal anti-DTX3L antibody (red) together with a rabbit polyclonal anti-ARTD9 antibody (green). Original magnification × 400. [file 1476-4598-13-125-S4.pdf]

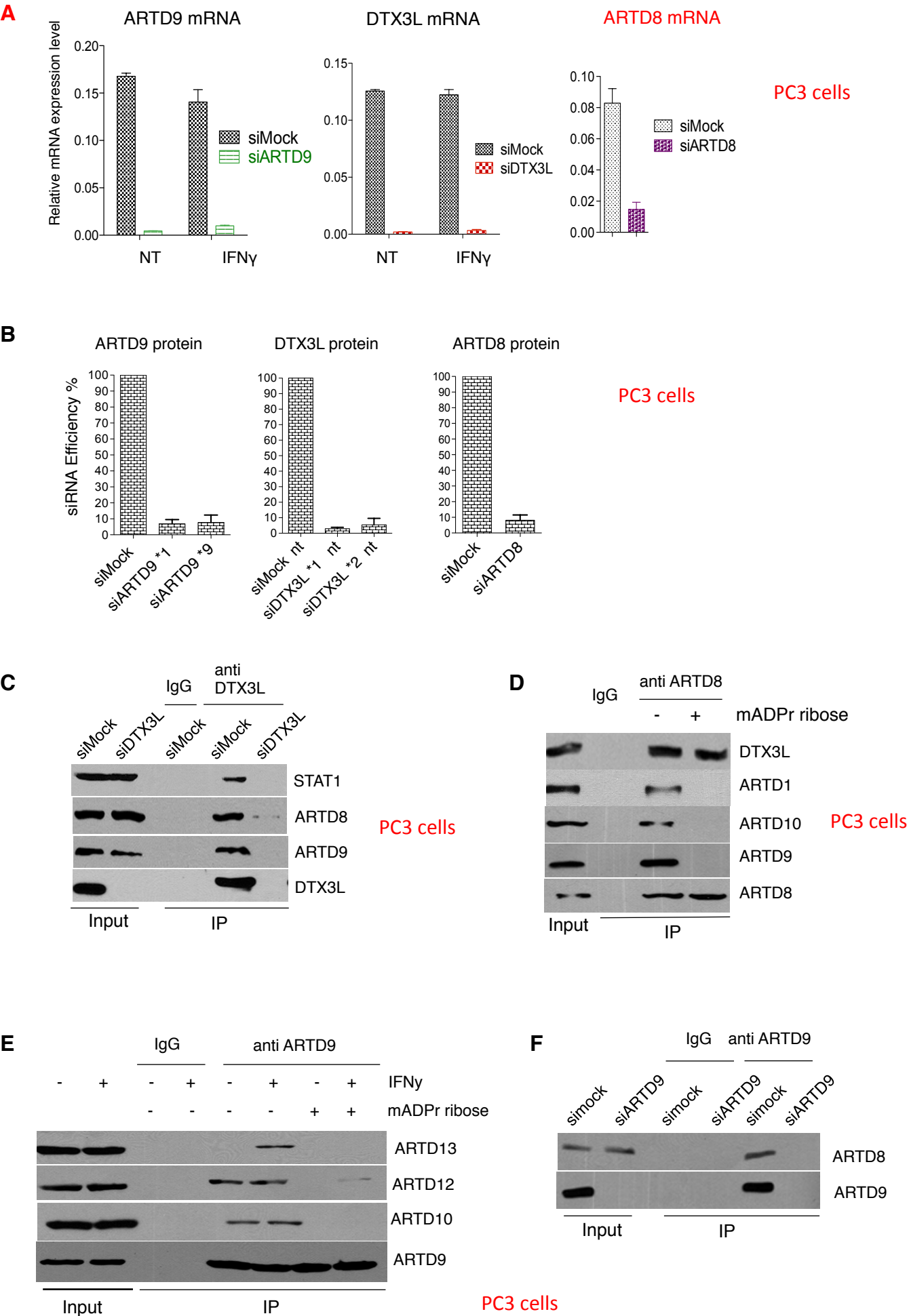

Supplement: Additional file 5: Figure S5 — Quantifications of ARTD8-, ARTD9- and DTX3L-siRNA knockdown efficiencies and analysis of ARTD8, ARTD9 and DTX3L containing complexes. (A and B) Analysis of ARTD8, ARTD9 and DTX3L-siRNA knockdown efficiency in PC3 cells. (A) Gene expression analysis of ARTD8, ARTD9 and DTX3L in PC3-siMock, PC3-siARTD8, PC3-siARTD9 and PC3-siDTX3L cells, respectively. ARTD8, ARTD9 and DTX3L mRNA levels were measured by RT-qPCR, normalized against GAPDH and presented as mean from three independent experiments performed in triplicate ± SE. (B) Quantification of ARTD8, ARTD9 and DTX3L protein levels in in PC3-siMock, PC3-siARTD8, PC3-siARTD9 and PC3-siDTX3L cells, respectively. ARTD8, ARTD9 and DTX3L levels were normalized to tubulin. Values represent the means of three independent experiments, and the error bars represent the SE. (C) Co-immunoprecipitation control analyses to confirm the specificity of the anti- DTX3L antibody. (D) Interactions of endogenous ARDT8 with ARTDs but not with DTX3L are mediated by (mono)-ADP-ribosylation. Endogenous ARTD8-ARTDx and ARTD8-DTX3L complexes from PC3 cell extracts were co-immunoprecipitated in presence or absence of 5 mM mono-ADP-ribose using epitope affinity purified anti-ARTD8 antibodies. Complexes were then separated on SDS PAGE, blotted and subsequently probed with antibodies against endogenous ARTD1, ARTD8, ARTD9, ARTD10 and DTX3L. ARTD1 was used as a positive control for ARTD8 and ARTD9 [80] and ARTD10 was used as a positive control for ARTD8 [44]. (E) Interactions of endogenous ARDT9 with ARTDs are mediated by (mono)-ADP-ribosylation. PC3 cells were stimulated for 1 h with IFNγ (200 U/ml) and endogenous ARTD9-ARTDx complexes subsequently co-immunoprecipitated in presence or absence of 5 mM mono-ADP-ribose using epitope affinity purified anti-ARTD9 antibodies. Complexes were then separated on SDS PAGE, blotted and subsequently probed with antibodies against endogenous ARTD9, ARTD10, ARTD12 (also known as PARP12) and ARTD13 (also known as PA [file 1476-4598-13-125-S5.pdf]

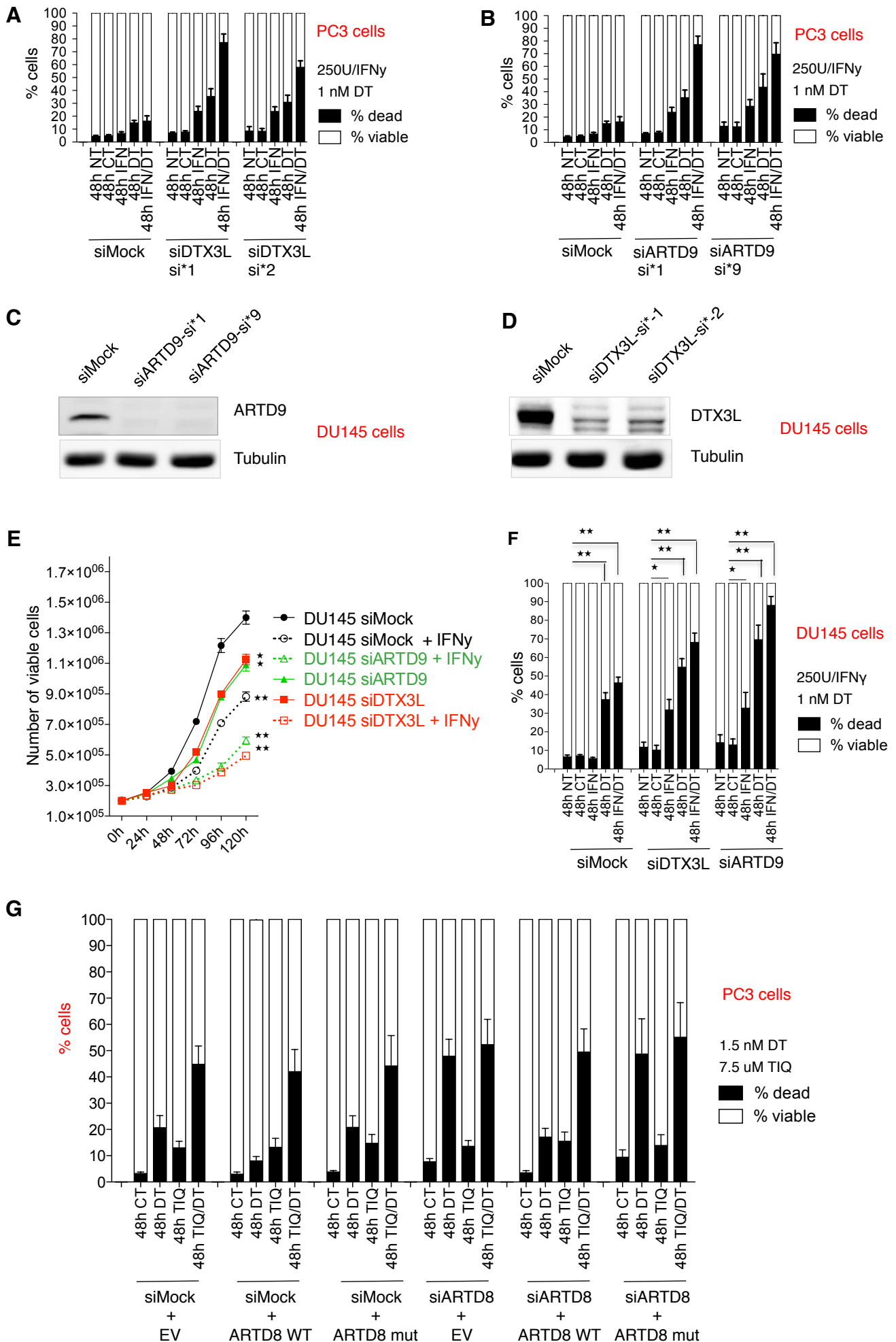

Supplement: Additional file 6: Figure S6 — Cell viability and proliferation analyses of siDTX3L, siARTD8 and siARTD9 single knockdown cells. (A and B) Cell viability analyses of PC3-siMock, PC3-siDTX3L cells silenced with si*1 or si*2 RNA oligos (A) and PC3-siARTD9 cells silenced with si*1 or si*9 RNA oligos (B) were assessed by the trypan blue exclusion assay. Cells were treated as indicated with IFNγ and/or docetaxel (DT) and counted after 48 h. Values represent the means of three independent experiments, and the error bars represent the SE. (C and D) Immunoblot analyses of ARTD9- and DTX3L-siRNA knockdown efficiencies in DU145 cells. Whole cell extracts were separated by SDS PAGE, blotted and subsequently probed with antibodies for ARTD9 (C), DTX3L (D) and tubulin. (E) Cell proliferation analyses of DU145-siMock, DU145-siDTX3L and DU145-siARTD9 single knockdown cells in presence or absence of IFNγ (200 U/ml) was assessed by the trypan blue exclusion assay. (F) Cell viability analyses of DU145-siMock, DU145-siDTX3L and DU145-siARTD9 knockdown cells were assessed by the trypan blue exclusion assay. Cells were treated as indicated with IFNγ and/or docetaxel (DT) and counted after 48 h, NT: not treated, CT: control treatment (solvent). (G) Survival of PC3-siMock or PC3- siARTD8 knockdown cells, complemented with non-degradable mouse cDNAs of active ARTD8 wild type or catalytically inactive ARTD8 mutant form, respectively, were assessed by the trypan blue exclusion assay. Cells were treated as indicated with docetaxel (DT) and/or with the ARTD inhibitor TIQ, CT: control treatment (solvent), EV: empty vector control. All Values shown in E to G represent the means of three independent experiments performed in triplicates, and the error bars represent the SE. Statistical analysis was performed using the Student's t test. *P < 0.05, **P < 0.001 and ***P < 0.0001. [file 1476-4598-13-125-S6.pdf]

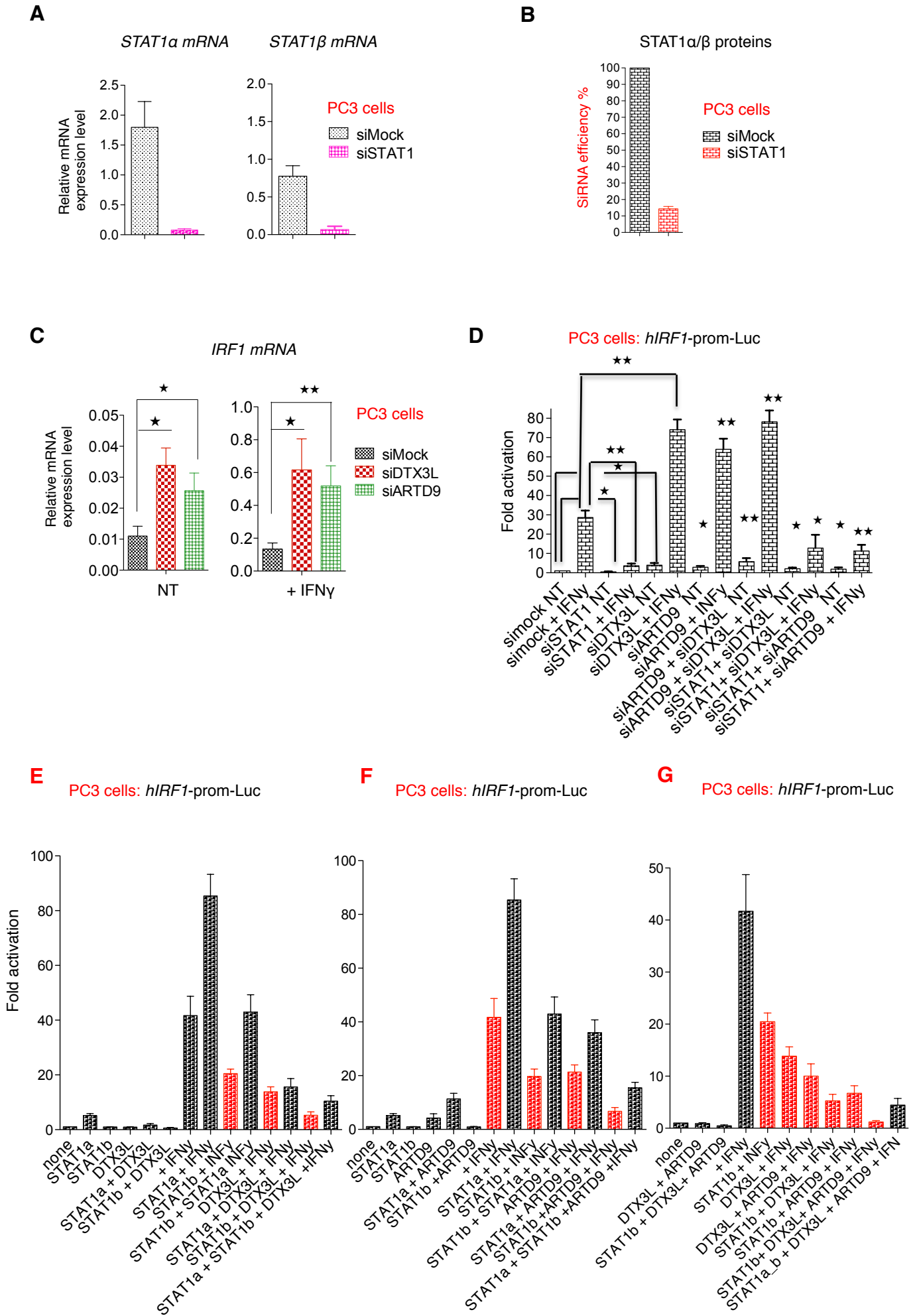

Supplement: Additional file 7: Figure S7 — Quantifications of STAT1-siRNA knockdown efficiencies and IRF1 promoter analysis (A and B). Analysis of STAT1-siRNA efficiency in PC3 cells. (A) Gene expression analysis of STAT1 in PC3-siMock and PC3-siSTAT1 knockdown cells; Total RNA was isolated from PC3-siMock and PC3-siSTAT1 knockdown cells and STAT1 mRNA levels were measured by RT-qPCR and normalized against GAPDH. (B) Quantification of STAT1 protein levels in PC3-siMock and PC3-siSTAT1 knockdown cells; STAT1 levels were normalized to tubulin. (C) Gene expression analysis of IRF1 in PC3-siMock, PC3-siARTD9 and PC3-siDTX3L knockdown cells. Total RNA was isolated from PC3-siMock, PC3-siARTD9 and PC3-siDTX3L knockdown cells and IRF1 mRNA levels were measured by RT-qPCR and normalized against GAPDH. All Values shown in A to C represent the means of three independent experiments, and the error bars represent the SE. (D) DTX3L- iRNA and ARTD9-siRNA mediated activation of the IRF1-promoter driven luciferase in PC3 cells. PC3 cells were co-transfected in series with mock-siRNA, STAT1-siRNA, DTX3L-siRNA or ARTD9-siRNA and plasmids for an IRF1-promoter-driven luciferase reporter vector as described in Material and Methods and subsequently treated with or without IFNγ (200 U/ml) for 4 h. (E-G) DTX3L and ARTD9 together with STAT1β inhibit the IRF1-promoter driven luciferase in PC3 cells. PC3 cells were co-transfected with an IRF1-promoter-driven luciferase reporter vector along with expression vectors for DTX3L, ARTD9 and/or STAT1α/β and subsequently treated with or without IFNγ (200 U/ml) for 4 h. IRF1-promoter-luciferase activities shown in D to G are presented as mean from five independent experiments performed in triplicates. The error bar represents the SE. Statistical analysis was performed using the Student's t test. *P < 0.05, **P < 0.001 and ***P < 0.0001. [file 1476-4598-13-125-S7.pdf]

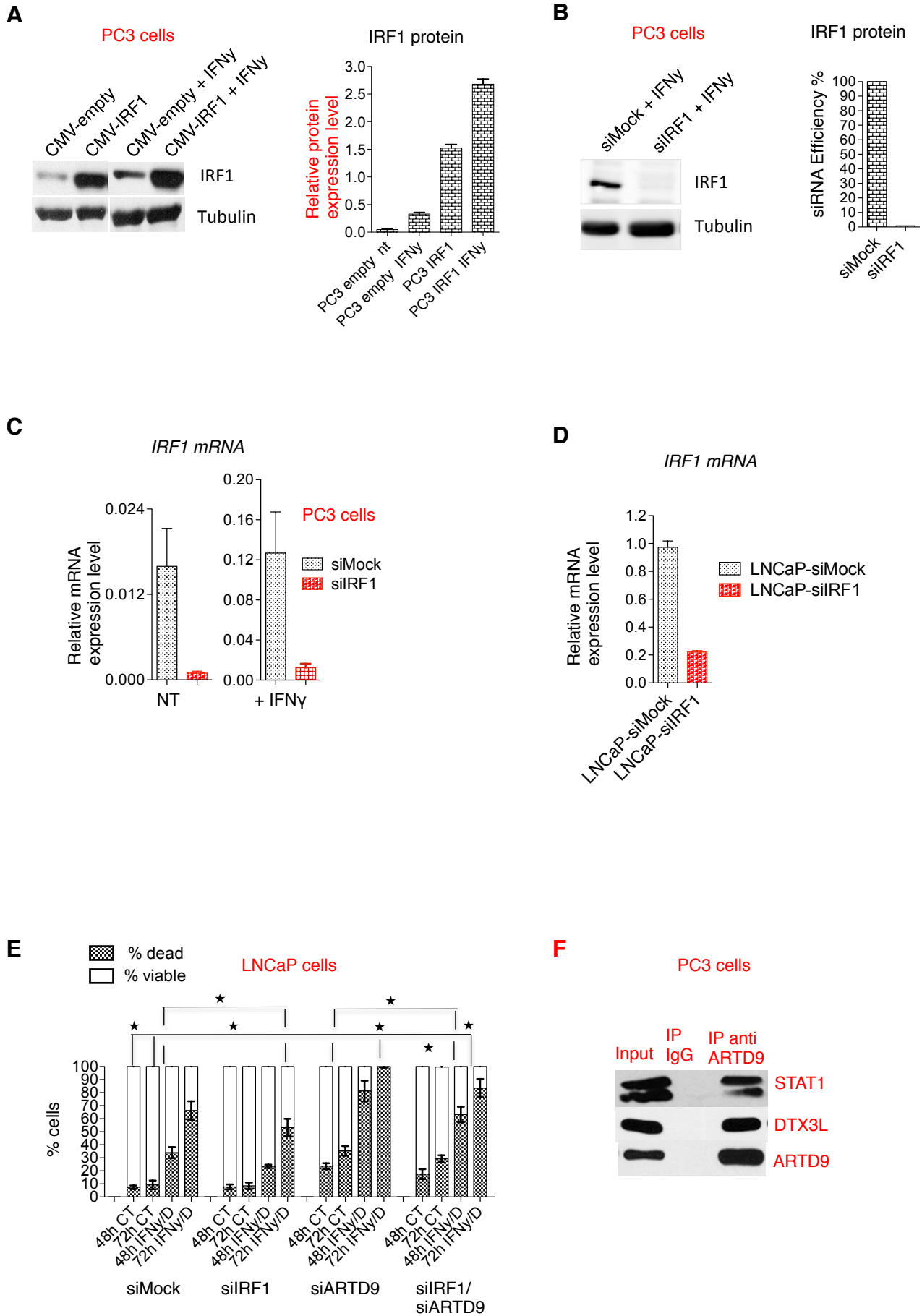

Supplement: Additional file 8: Figure S8 — Quantifications of IRF1 protein levels, quantifications of IRF1-siRNA knockdown efficiencies and cell viability analysis of siIRF1 knockdown cells. (A) Immunoblot analyses of IRF1 protein levels in PC3-CMVprom-empty-control and PC3-CMVprom-IRF1 cells. Whole cell extracts of PC3-CMVprom-empty-control and PC3-CMVprom7 IRF1 cells were separated by SDS PAGE, blotted and probed with antibodies for IRF1 and tubulin. (A right panel) Quantification of IRF1 protein levels in PC3-CMVprom-empty-control and PC3-CMVprom-IRF1 cells; IRF1 levels were normalized to tubulin. (B and C) Analysis of IRF1- siRNA efficiency in PC3 cells. (B) Immunoblot analyses of IRF1 protein levels in PC3-siMock and PC3-siIRF1 cells. Whole cell extracts of PC3-siMock and PC3-siIRF1 cells were separated by SDS PAGE, blotted and probed with antibodies for IRF1 and tubulin. (B right panel) Quantification of IRF1 protein levels in PC3-simock and PC3-siIRF1 cells; IRF1 levels were normalized to tubulin. (C) Gene expression analysis of IRF1 in PC3-siMock and PC3-siIRF1 knockdown cells. IRF1 mRNA levels were measured by RT-qPCR and normalized against GAPDH. (D) Gene expression analysis of IRF1 in LNCaP-siMock and LNCaP-siIRF1 knockdown cells. IRF1 mRNA levels were measured by RT-qPCR and normalized against GAPDH. (E) Cell viability analyses of PC3-siMock, PC3-siIRF1, PC3-siARTD9 and PC3-siARTD9/siIRF1 cells were assessed by the trypan blue exclusion assay. Cells were treated as indicated with 50 ng/ml IFNγ and 0.25 μM doxorubicin (D) and counted after 48 h and 72 h, respectively, NT: not treated, CT: control treatment (solvent). All values shown in A to E represent the means of three independent experiments, and the error bars represent the SE. Statistical analysis was performed using the Student's t test. *P < 0.05, **P < 0.001 and ***P < 0.0001. (F) Co-immunoprecipitation analyses of endogenous nuclear DTX3L/ARTD9/STAT1 complexes, respectively in PC3 cells. Endogenous STAT1, DTX3L or RTD9 complexes were co- [file 1476-4598-13-125-S8.pdf]

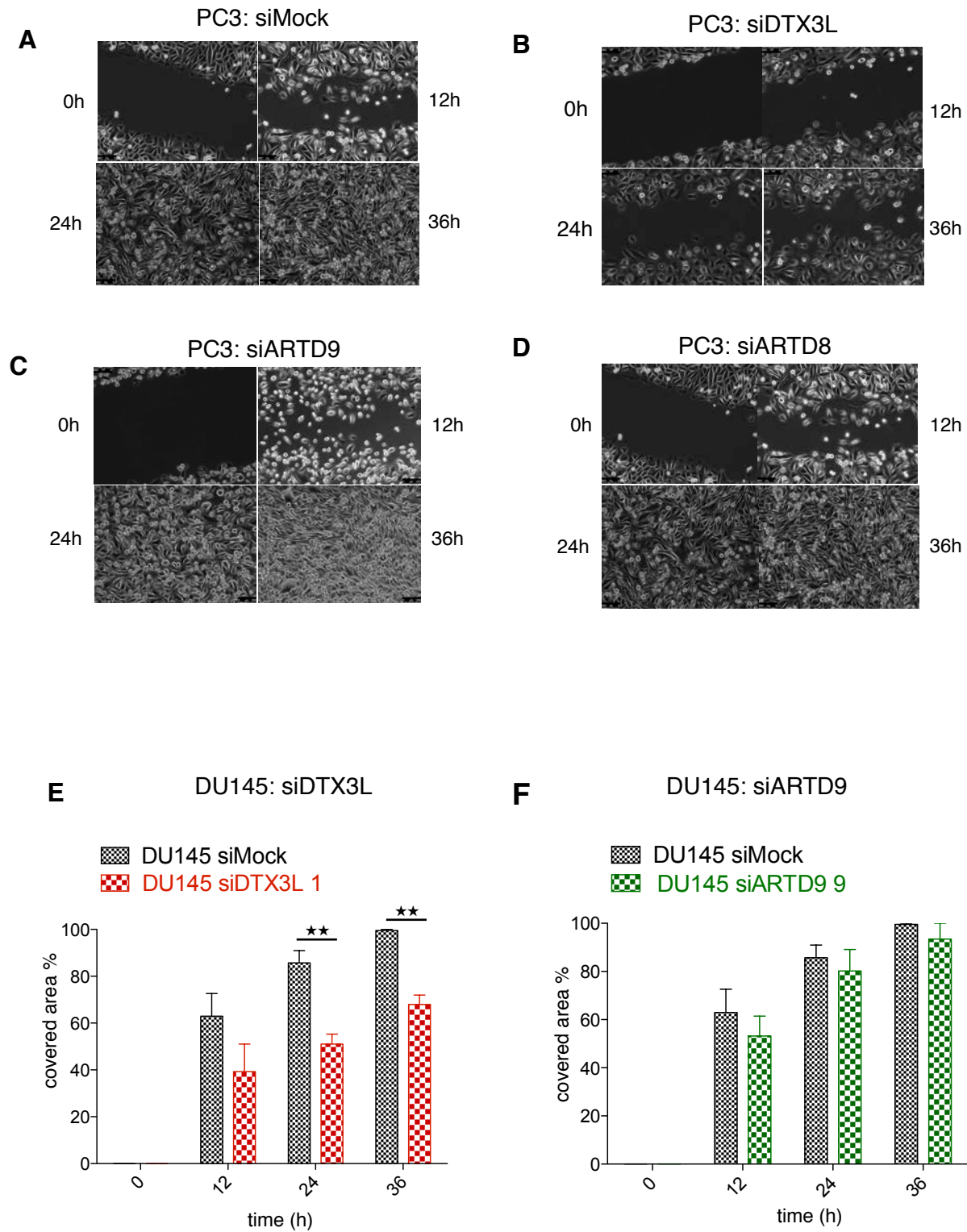

Supplement: Additional file 9: Figure S9 — Photographs and quantification of cell migration in siARTD8, siARTD9 and siDTX3L single knockdown prostate cancer cells. (A-D) Photographs of cell migration in PC3 prostate cancer cells. PC3-siMock (A), PC3-siDTX3L (B), PC3-siARTD9 (C) and PC3-siARTD8 (D) single knockdown cells were seeded into 6-well plates and treated as described in Material and Methods. At 0, 12, 24, and 36 h photographs were made. (E and F) Quantification of cell migration in DU145 prostate cancer cells. DU145-siDTX3L (E) and DU145-siARTD9 (F) single knockdown cells were seeded into 6-well plates and treated as described in Material and Methods. At 0, 12, 24, and 36 h photographs were made and quantified as described in Material and Methods. Values represent the mean of three independent experiments and the error bars represent the SE. Statistical analysis was performed using the Student's t test. *P < 0.05, **P < 0.001 and ***P < 0.0001. [file 1476-4598-13-125-S9.pdf]

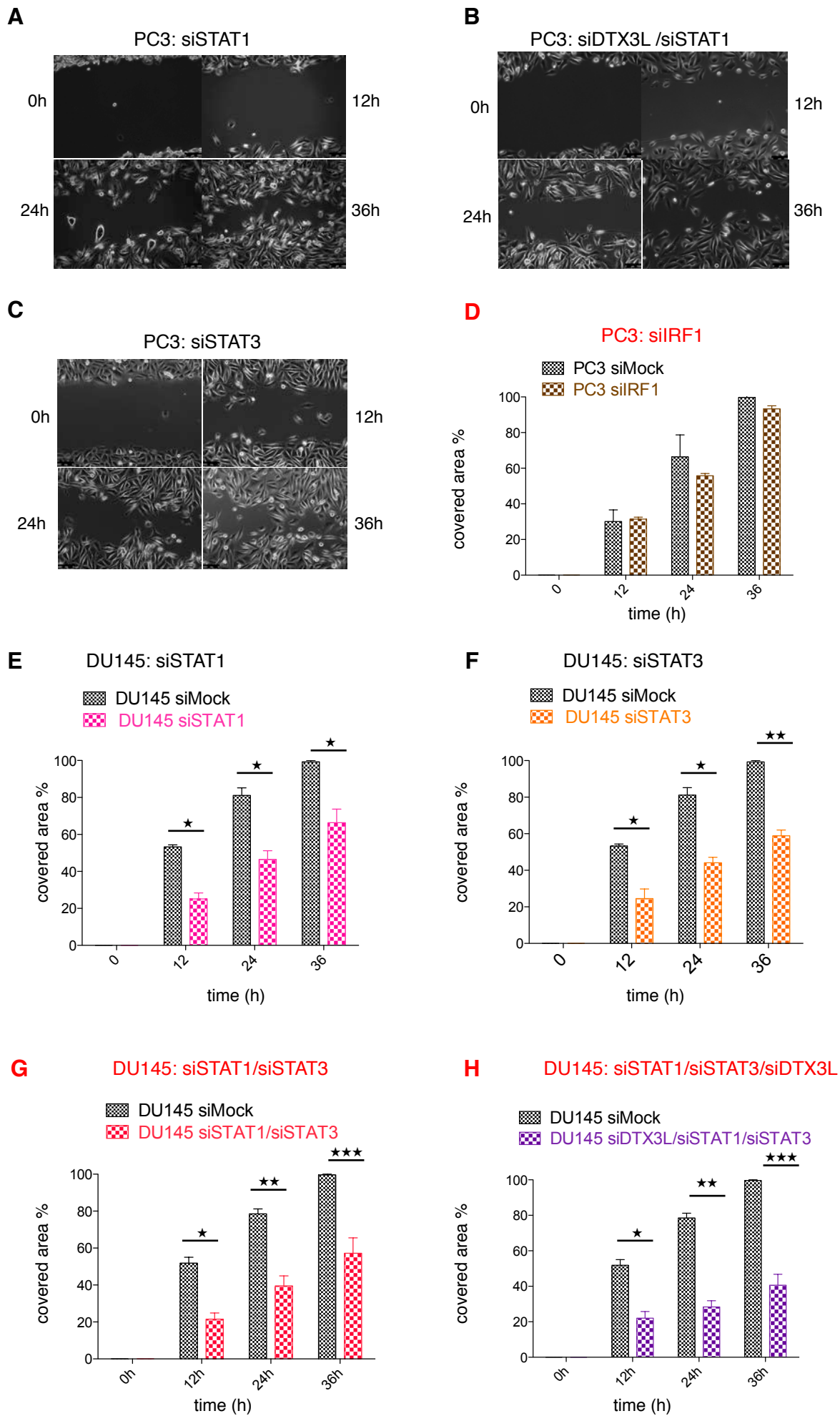

Supplement: Additional file 10: Figure S10 — Photographs and quantification of cell migration in single, double and triple knockdown prostate cancer cells. (A-C) Photographs of cell migration in PC3 prostate cancer cells. PC3-siSTAT1 (A) PC3-siDTX3L/siSTAT1 (B) and PC3-siSTAT3 (C) single knockdown cells were seeded into 6-well plates and treated as described in Material and Methods. At 0, 12, 24, and 36 h photographs were made. (D-F) Quantification of cell migration in PC3 and DU145 prostate cancer cells. PC3-siIRF1 (D), DU145-siSTAT1 (E) and DU145-siSTAT3 (F) single knockdown cells, DU145-siSTAT1/siSTAT3 (G) double knockdown cells and DU145-siDTX3L/siSTAT1/siSTAT3 (H) triple knockdown cells were seeded into 6-well plates and treated as described in Material and Methods. At 0, 12, 24, and 36 h photographs were made and quantified as described in Material and Methods. Values represent the mean of three independent experiments and the error bars represent the SE. Statistical analysis was performed using the Student's t test. *P < 0.05, **P < 0.001 and ***P < 0.0001. [file 1476-4598-13-125-S10.pdf]
