# Supplementary material for: DTX3L and ARTD9 inhibit IRF1 expression and mediate in cooperation with ARTD8 survival and proliferation of metastatic prostate cancer cells
Source: Mol Cancer. 2014 May 27;13:125. doi: 10.1186/1476-4598-13-125 (PMC4070648; doi:10.1186/1476-4598-13-125)
Supplement: Additional file 11: Table S1 — siRNA sequences. [file 1476-4598-13-125-S11.doc]

**Table S1: siRNA sequences**

| **siRNA** | **Targeting sequence** |
| --- | --- |
| AllStar mock siRNA negative control | GGGUAUCGACGAUUACAAA |
| ARTD9-shRNA #-1 | TGCAGGTTCTAAAGGTGGA |
| ARTD9-shRNA #-2 | GGCAAAGTCAATTCTACAA |
| ARTD9-siRNA #-9 | TTACCTTGGGTGAACTAAC |
| ARTD9-siRNA #-10 | GGACAGAGTTAGAGATTGAAAC |
| STAT1-siRNA #-6 (validated by Qiagen) | CAGAAAGAGCTTGACAGTAAA |
| STAT1-siRNA #-7 (validated by Qiagen) | CCAGATGTCTATGATCATTTA |
| ARTD8-shRNA #-1 | CTAGTGCAGATGTGTATAA |
| ARTD8-shRNA #-2 | GGA AAG GGC TCA CTC ACA ATT |
| DTX3L-shRNA #-1 | TCCAGGTTATGAGTCCTTTGGCA |
| DTX3L-shRNA #-2 | GTTAGAGGTGGGTCCGAAATAA |
| DTX3L-shRNA #-3 | GGCAAGCATTGGTAATAAATGGA |
| DTX3L-shRNA #-4 | GCCCTGCCACAGTAATGCTATA |
| STAT3-siRNA #-7 (validated by Qiagen) | CAGCCTCTCTGCAGAATTCAA |
| STAT3-siRNA #-8 (validated by Qiagen) | CAGGCTGGTAATTTATATAAT |
| IRF1-siRNA #-1 | CCAAGAACCAGAGAAAAGA |
| IRF1-siRNA #-2 | AGACCAGAGCAGGAACAAG |
| JAK1-siRNA #-1 (validated by Qiagen) | CTGGGAATTCCAACCATCCAA |
| JAK1-siRNA #-6 (validated by Qiagen) | CACGGATAACATCAGCTTCAT |
