# Supplementary material for: DTX3L and ARTD9 inhibit IRF1 expression and mediate in cooperation with ARTD8 survival and proliferation of metastatic prostate cancer cells
Source: Mol Cancer. 2014 May 27;13:125. doi: 10.1186/1476-4598-13-125 (PMC4070648; doi:10.1186/1476-4598-13-125)
Supplement: Additional file 12: Table S2 — qPCR Primer. [file 1476-4598-13-125-S12.doc]

**Table S2: qPCR Primer**

| **Target gene(human)** | **FWD Primer** | **Rev Primer** |
| --- | --- | --- |
| ARTD8 | GACTGTCGCTATGTGCTTCAC | GGACAAGCTCTCAGTGATCTCC |
| ARTD9 | GGCAAAGAGGTCCAAGATGCTG | GCCTCACACATCTCTTCCACGT |
| DTX3L | CCAGGTTATGAGTCCTTTGGCAC | TGCAGTTCGCTGTATTCCAGGG |
| STAT1α | TGCTGTTACTTTCCCTGACATCA | GAGTAGCAGGAGGGAATCACAGAT |
| STAT1β | CGCAATTACAAAGTCATGGCTG | AGCAAGGCTGGCTTGAGGTTT |
| IRF1 | AAAAGGAGCCAGATCCCAAGA | CATCCGGTACACTCGCACAG |
| STAT3 | CATCATGGGCTTTATCAGTAAGGA | GTCAATGGTATTGCTGCAGGTCGT |
| GAPDH | GAAATCCCATCACCATCTTCC | GAGCCCCAGCCTTCTCCATG |
